# Extracellular vesicles originating from induced pluripotent stem cell-derived chondrocytes facilitate the regeneration of osteoarthritic cartilage

**DOI:** 10.1016/j.jot.2025.101035

**Published:** 2026-01-10

**Authors:** Yaqian Hu, Pengzhen Cheng, Liyuan Jia, Xue Hao, Guangyu Ding, Meige Han, Jing Fan, Weiguang Lu, Chuxin Zhou, Zhuojing Luo, Liu Yang

**Affiliations:** a Department of Orthopedics, Xijing Hospital, The Fourth Military Medical University, Xi'an, 710032, China; b Pediatric Orthopaedic Hospital, Honghui Hospital, Xi'an Jiaotong University, Xi'an, 710021, China; c Xi'an Key Laboratory of Skeletal Developmental Deformity and Injury Repair, Xi'an, 710021, China; d College of Life Sciences, Northwest University, Xi'an, 710069, China; e Medical Research Institute, Northwestern Polytechnical University, 710072, Xi'an, China

**Keywords:** Small extracellular vesicles, Induced pluripotent stem cells, Chondrocytes, Cartilage regeneration, Proteome

## Abstract

**Background:**

Articular cartilage, a highly specialized avascular connective tissue, forms a smooth, load-bearing surface on articulating bone ends to facilitate frictionless joint movement. While cell-derived small extracellular vesicles (sEVs) have emerged as promising therapeutic biomaterials for cartilage regeneration, two major limitations impede their clinical translation: the scarcity of autologous donor cells, and suboptimal functional efficacy of conventional sEVs preparations.

**Methods:**

We established an induced pluripotent stem cell (iPSC)-based approach by differentiating iPSCs into chondrocytes (designated iChondrocytes). After characterizing the derived iChondrocytes, we isolated their secreted sEVs (iChondrocyte-sEVs) and systematically evaluated their therapeutic effects and underlying mechanisms in cartilage regeneration through *in vitro* and *in vivo* experiments.

**Results:**

Functional assays revealed that iChondrocyte-sEVs significantly enhanced chondrogenic differentiation, stimulated extracellular matrix (ECM) deposition, and maintained chondrocyte homeostasis. In murine osteoarthritis (OA) models, intra-articularly administered iChondrocyte-sEVs efficiently penetrated deep cartilage layers, alleviated OA-associated pathological changes, and restored cartilage microstructure and biomechanical properties to near-normal levels. Furthermore, therapeutic intervention with iChondrocyte-sEVs resulted in significant improvements in joint mobility and motor function in OA-affected mice. Proteomic profiling identified serine racemase (SRR) as a key upregulated protein in iChondrocyte-sEVs. Mechanistic studies suggested that SRR may contribute to cartilage regeneration by suppressing the P38/ERK signaling pathway.

**Conclusions:**

This study highlights the significant therapeutic potential of iChondrocyte-sEVs, demonstrating their ability to promote both structural and functional regeneration of articular cartilage. These findings suggest a promising novel strategy for OA treatment.

**The translational potential of this article:**

This study highlights the therapeutic potential of iChondrocyte-sEVs, demonstrating their ability to promote both structural and functional regeneration of articular cartilage. These findings indicate a clinical conversion potential for OA treatment.

## Graphical abstract

**Figure:**
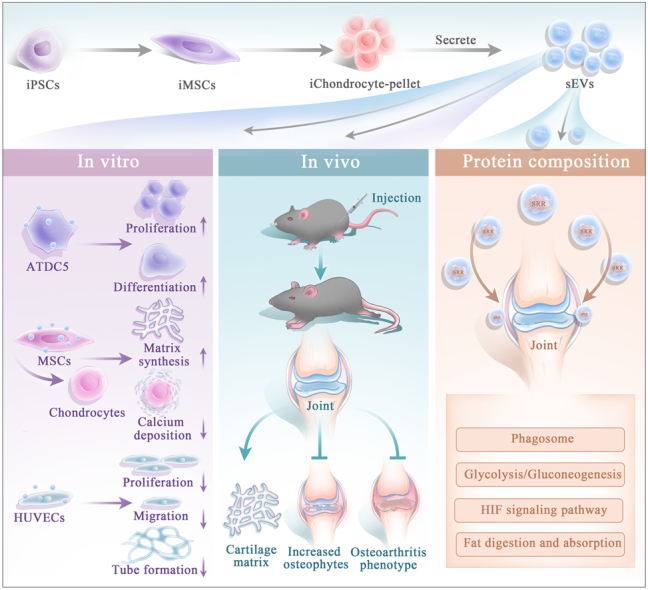

## 1 Introduction

Articular cartilage, which covers the ends of bones in articulating joints, facilitates smooth and frictionless joint movement in humans and animals [1]. Cartilage-related tissue defects, commonly resulting from trauma, degenerative diseases, and age-related changes, represent a significant cause of musculoskeletal disability. The inherent avascular nature and low cellular density of cartilage tissue contribute to its limited regenerative capacity [2], often leading to progressive joint degeneration and the development of OA, which can ultimately result in significant functional impairment. In the context of cell therapy for OA, the utilization of stem cells as cellular sources for cartilage regeneration holds promising potential [3]; however, challenges may arise in terms of the collection, transportation, and potential immune responses associated with stem cells [4]. Recently, there has been a growing interest in the use of sEVs for repairing cartilage damage as part of the advancement in cell-free therapy [5]. The sEVs are diverse, membrane-bound structures originating from cells, with a size range of 50–150 nm. They play a crucial role in facilitating intercellular communication by transferring their contents (lipids, nucleic acids and proteins) under both normal physiological and pathological conditions [6]. Generally, sEVs exhibit minimal side effects, possess characteristics such as non-tumorigenicity and high targeting ability, and are generally well tolerated with low immunogenicity and toxicity [7].

The function of sEVs is analogous to that of their parent cells [8]. Mesenchymal stem cell (MSC)-sEVs exhibited effects on enhancing chondrocyte functionality and facilitating articular cartilage repair [9,10]; however, they also induced hypertrophic differentiation accompanied by vascular ingrowth within cartilage progenitor cell-engineered constructs [11], this was detrimental to the sustained maintenance of cartilage homeostasis. The role of chondrocyte-sEVs in cartilage repair has been preliminarily investigated, they have been found to increase the expression of chondrogenic proteins SOX9 and ACAN, thereby contributing to the maintenance of a healthy cartilage phenotype [12]. However, the dosage of EVs should be taken into consideration when utilizing them for cartilage repair, particularly in cases of severe or extensive cartilage damage. The limited proliferative capacity of articular chondrocytes poses a challenge in obtaining an adequate number of chondrocytes for the production of a substantial quantity of sEVs required for cartilage repair. In recent years, iPSCs [13] have emerged as a promising cell source in tissue engineering due to their unlimited proliferation and differentiation potential. iPSCs possess similar pluripotency to embryonic stem cells (ESCs), but without the issues of immune rejection and ethical concerns. IPSCs can be differentiated into various cell phenotypes, including chondrocytes. There are numerous methodologies available for differentiating iPSCs into chondrocytes [14].

The proteins, RNAs, and lipids components carried by sEVs determine their functional role [15]. sEVs cargo exhibit diversity and functional complexity, participating in various biochemical and cellular processes [16]. This wide range of biological activities endows sEVs with the ability to induce diverse cellular responses and interact with different cell types [17]. The presence of multiple effector components in sEVs is essential, and the proteomic features of sEVs provide valuable insights into their potential roles [18]. Therefore, comprehending the protein composition and underlying biological processes of sEVs can significantly enhance our understanding of the mechanisms involved in sEVs-mediated articular cartilage repair.

In this study, the iPSCs were induced to undergo chondrogenic differentiation, resulting in the acquisition of iPSCs-derived chondrocytes (iChondrocytes). We characterized the iChondrocyte-sEVs and assessed their impact on cell proliferation, migration, differentiation, and extracellular matrix deposition. The regenerative potential of iChondrocyte-sEVs for articular cartilage repair was evaluated in OA mice model. Finally, we identified the proteome of iChondrocyte-sEVs and elucidated the protein subsets and associated signaling pathways involved in their functionality. This study systematically and comprehensively elucidates the significant role of iChondrocyte-sEVs in facilitating cartilage repair. As a biomaterial with properties closely resembling those of natural chondrocyte-sEVs, iChondrocyte-sEVs offered a promising therapeutic strategy for articular cartilage regeneration.

## 2 Materials and methods

### 2.1 Induction and identification of iChondrocytes from iPSCs

The induction of iChondrocytes was conducted based on established classical techniques [19]. In brief, human iPSCs were established with the help of Shenzhen Cell Inspire Biotechnology Company [20] and cultured with mTeSR Plus™ medium (STEMCELL Technologies, Canada). For the identification of iPSCs, the cell colonies were stained with ALP detection kit (Beyotime, China), and the cells were immunofluorescently stained using antibodies against NANOG and OCT4 (Abcam, UK). When the cell fusion rate exceeds 80 %, use Cell Dissociation Buffer to digest the cells and resuspend them in a 1:3 ratio in a culture dish. After the cells adhere to the wall, replace the culture medium with MSCs induction medium. When the cell fusion rate exceeds 80 %, subculture at a ratio of 1:2. When the passage reaches P4, collect the induced MSCs (iMSCs) and perform flow cytometry identification. After passing the identification, iMSCs were added to the center of a 24-well plate at a concentration of 300,000/20ul, and left to rest in a cell incubator for 3 h. After the cells had coalesced, 0.7 ml of chondrogenic differentiation medium (SclenCell, USA) was gently added. After 2 days, the cells formed micro-pellets, and the medium was changed again. Afterwards, the medium was changed every other day, and the iChondrocyte-pellets and the supernatants were collected from day 36∼50.

### 2.2 Extraction and characterization of sEVs

The cell supernatant was centrifuged at 300*g* for 10 min at 4 °C to eliminate dead cells. The resulting supernatant was then transferred to a fresh centrifuge tube and subjected to centrifugation at 2000 g for 10 min at 4 °C to remove large microvesicles. Subsequently, the supernatant was collected and further centrifuged at 10,000g for 30 min at 4 °C to eliminate cell debris. The supernatant was subsequently transferred into ultra-high speed centrifuge tubes and subjected to centrifugation at 110,000g for 75 min at 4 °C (Optima™ L-90k Ultracentrifuge, Beckman Coulter, USA). The resulting supernatant was discarded, while the precipitate was resuspended in a small amount of PBS, filtered through a membrane with a pore size of 0.22 μm, and transferred into another ultra-high speed centrifuge tube. It underwent another round of centrifugation under conditions of 4 °C and force of 110,000g for 75 min before discarding the supernatant. Finally, the precipitate was resuspended in a minimal volume of PBS, divided into aliquots, and stored at −80 °C until use. Nanoparticle tracking analysis (NTA) performed on a NanoSight NS500 instrument (ZetaVIEW S/N 17–310, PARTICLE METRIX) analyzed the particle size and numbers of sEVs. The morphology of sEVs was verified by transmission electron microscopy (Tecnai G2 Spirit BioTwin, FEI).

### 2.3 Proliferation assay

Briefly, cells were seeded in 96-well plates at a density of 5000 cells per well and grown overnight at 37 °C in a humidified incubator with 5 % CO_2_.Then, cells were treated with PBS or sEVs (10^9^ particles/mL, the concentration of sEVs used for *in vitro* experiments was selected based on preliminary dose–response studies). After hatching at day 1, 2, 3 and 4, 10 μL CCK-8 solution (Beyotime, China) and 100 μL fresh medium were added to each well. The cells were incubated for 2 h at 37 °C. The absorbance (OD) value was measured at 450 nm using a fully automatic microplate Reader (Bio-Tek, USA). All experiments were performed in five repeats.

### 2.4 Chondrogenic differentiation of ATDC5

ATDC5 was seeded in 24-well plates supplemented with DMEM/DF12 + 5 % Fetal bovine serum, followed by stimulation with different groups of sEVs (iPSCs, iMSCs, and iChondrocytes) at a density of 10^9^ particles/mL, and the medium was changed every 2 days. On day 14, cells were fixed for safranin O and alcian blue staining.

### 2.5 Formation of chondrocyte-pellets derived from MSCs

Human MSCs were dotted with 4 × 10^5^ cells per well in the center of a single well of a 24-well plate in a volume of 20 μL. The cells were placed in a cell incubator and adhered for 2.5h to form pellets. The wells were gently supplemented with 0.7 ml chondrogenic medium (HUXMX-90041, Cyagen, China) with 1 × 10^9^ particles/ml sEVs. Subsequently, the culture plates were incubated overnight. During *in vitro* differentiation for 21 days to obtain chondrocyte-pellets, the medium was replaced every 2 days.

### 2.6 Histopathological assay

The 5-μm sections were deparaffinized with xylene and rehydrated. Subsequently, 3 % H_2_O_2_ was added to block endogenous peroxidase activity. Antigen repair was then performed with 0.1 % trypsin and blocking was performed with goat serum. Subsequently, the sections were sequentially incubated with the primary antibody overnight at 4 °C and the secondary antibody at 37 °C for 1 h. Finally, horseradish peroxidase-labeled streptavidin-biotin was added to observe the immunoreactivity under an optical microscope.

### 2.7 Immunofluorescent staining

At day 21, the induced chondrocyte-pellets of each group were frozen sectioned. The primary antibodies were COLII (28459-1-AP, Proteintech, USA), ACAN (AB1031, Millipore, USA), SOX9 (ab185966, Abcam, UK), COLI (ab34710, Abcam, UK), COLX (A18604, Abclonal, China), and the secondary antibodies were red/green fluorescent secondary antibodies. The slices were sealed and stored at −20 °C for immunofluorescence imaging (FV 3000, Olympus, Japan).

### 2.8 OA chondrocytes culture and treatment

Mice primary chondrocytes were seeded in 6-well cell plates at a density of 2 × 10^6^ cells/well and supplemented with 2.5 ml DMEM low glucose medium (11885084, Gibco, USA) with 10 % Fetal bovine serum. After 5 days, the chondrocytes were treated with 10 ng/ml IL-1β (PMC0815, Thermo Fisher Scientific, USA) with or without 1 × 10^9^ particles/ml iChondrocyte-sEVs for 3 days. Then chondrocytes were fixed using 4 % paraformaldehyde and subsequently stained with Safranin O and Alcian Blue.

### 2.9 Experimental OA mice model

All animal experimental procedures were approved by the Ethics in Experimental Animal Center of the Fourth Military Medical University (permission code IACUC-20230023). Destabilization of the medial meniscus (DMM) surgery was performed on the knee of 8-week-old C57BL/6J male mice. Mice were anesthetized via inhalation of 3 % isoflurane (in 100 % oxygen) using an induction chamber, followed by maintenance at 1.5 % isoflurane delivered via a nose cone. Prior to anesthesia, preemptive analgesia (buprenorphine, 0.1 mg/kg) was administered. Depth of anesthesia was confirmed by the absence of toe pinch reflex and stable respiratory rate. Body temperature was maintained at 37 ± 0.5 °C using a feedback-controlled heating pad. The sham group included mice that underwent solely medial capsulotomy. Four weeks after surgery, sEVs (8 μl per knee, 1 × 10^10^ particles/ml) were injected into the joint cavity once a week for 4 weeks. Mice were euthanized via gradual CO_2_ inhalation (displacement rate 20 %/min). All animal experiments complied with the ARRIVE guidelines and were carried out in accordance with the U.K. Animals (Scientific Procedures) Act, 1986 and associated guidelines, EU Directive 2010/63/EU for animal experiments.

### 2.10 Micro-structure analysis with SEM

The joints of mice in each group were trimmed with small scissors, and only cartilage and bone were retained. The joints were placed in electron microscope fixative solution at 4 °C overnight, lyophilized, and glued to the metal base. The cartilage was facing up and electron microscopy (Quattro S, Thermo Fisher Scientific, USA) was performed, and images were acquired at 500X and 30000× magnification when each sample was scanned.

### 2.11 Micro computed tomography (uCT)

After the mice were sacrificed, the joints of the mice were fixed with 4 % paraformaldehyde for 3 days, and uCT scanning (SKYScan, BRUKER, Belgium) was performed. The images obtained from the scanning were analyzed by CTAn and CTVox software.

### 2.12 Young's modulus assay

After the joints were separated, the femoral condyle was glued to the petri dish. The nanoindentation instrument (Piuma, OPTICS 11, Netherlands) was used to press the probe on the trochlea. The mechanical strength of the probe was 47.9N/m and the tip diameter was 25.5um. Mechanical information was collected 24 sites from 6 mice per group.

### 2.13 Gait analysis

The gait of the mice was detected using Digigait™ imaging system (Mouse Specifics, Inc). After sEVs injection for 4 times, all mice were first trained on the running belt, and the speed of the running belt was gradually increased until 20 cm/s, so that all mice could adapt to running. Then each mouse was put on the running belt in turn, the speed was constant at 20 cm/s, and the effective running time was collected for 3∼5s. The data of each group were analyzed.

### 2.14 Western blot analysis

Western blot was carried out as previously published [21]. Cells and isolated sEVs were lysed with lysis buffer (P0013, Beyotime Biotechnology, China). After the measurement of protein concentration, the solution was mixed with 5 × SDS-PAGE loading buffer (Beyotime Biotechnology, China) and boiled at 95 °C for 10 min. Proteins were separated with 10 % or 8 % SDS-PAGE gel and transferred to polyvinylidene difluoride (PVDF) membranes. Afterwards, the PVDF membranes were blocked with 5 % skim milk (P0216, Beyotime Biotechnology, China) for 1h and then incubated with a primary antibody solution at 4 °C overnight. The following primary antibodies were used for western blot analysis: CD9 (RGAB101, Rengen Biosciences, China), CD63 (RGAB103, Rengen Biosciences, China), Alix (RGAB100, Rengen Biosciences, China), SRR (17955-1-AP, Proteintech, China), SOX9 (GB11280, Servicebio, China), COLII (28459, Proteintech, China), ACAN (68350, Proteintech, China), β-actin (GB15003, Servicebio, China), SynGAP (19739, Proteintech, China), JNK1 (A23206, Abclonal, China), P-JNK (AP1337, Abclonal, China), P38 (GB154685, Servicebio, China), P-P38 (GB153380, Servicebio, China), ERK (4695, CST, USA), and P-ERK(4370, CST, USA). PVDF membranes were incubated with the primary antibodies overnight at 4 °C. Then, after PVDF membrane was washed using TBST for three times, HRP-conjugated secondary antibodies were applied for incubation for 1 h at room temperature. Chemiluminescent signals were visualized by the ECL Western Blot detection kit and imaging system. All experiments were repeated at least 3 times.

### 2.15 Statistical analysis

All data were analyzed using GraphPad Prism software. Results are presented as the mean ± standard deviation (SD). Statistical comparisons between data sets were conducted with an analysis of normality and variance, followed by a two-tailed unpaired Student's t test for two group comparisons and one-way ANOVA with Turkey's post-hoc tests for multiple group comparisons. ∗*P* < 0.05, ∗∗*P* < 0.01, and ∗∗∗*P* < 0.001 were considered statistically significant.

## 3 Results

### 3.1 Characterization of iPSCs, iChondrocytes, and sEVs

The iPSCs exhibited characteristic embryonic stem cell-like morphology (Fig. S1a) and demonstrated robust alkaline phosphatase (ALP) activity, as evidenced by intense positive staining (Fig. S1b). Immunofluorescence analysis confirmed the expression of key pluripotency markers, NANOG and OCT4 (Fig. S1c and d), thereby validating their pluripotent state. Following induction of mesenchymal differentiation, the iPSCs successfully gave rise to induced mesenchymal stem cells (iMSCs) that morphologically resembled primary MSCs (Fig. S1e). The trilineage differentiation potential of iMSCs was subsequently confirmed through their capacity to undergo osteogenic (Fig. S1f), chondrogenic (Fig. S1g), and adipogenic (Fig. S1h) differentiation. Flow cytometric characterization revealed a surface marker profile consistent with conventional MSCs, with high expression levels of CD105 (98.9 %) and CD73 (99.2 %), and minimal expression of hematopoietic markers CD34 (2.0 %), CD45 (1.0 %), and HLA-DR (1.6 %) (Fig. S1i).

**Fig. 1:**
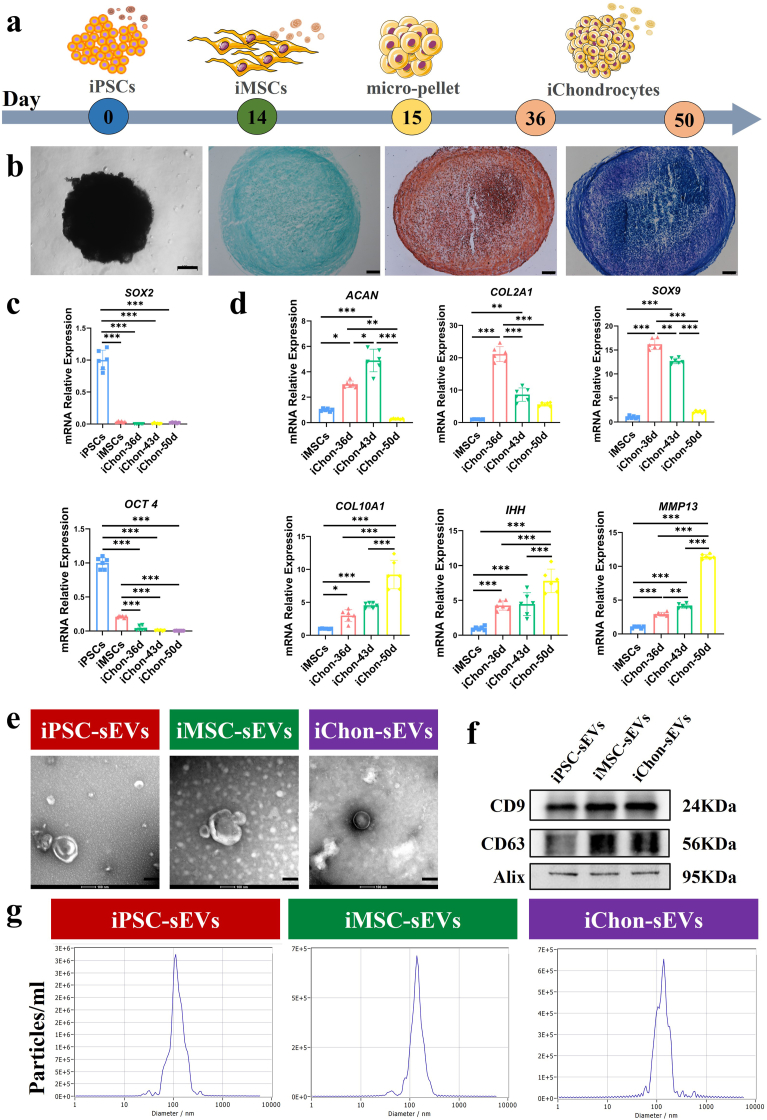
Characterization of iChondrocytes and derived sEVs. **a** Schematic representation of the differentiation protocol from iPSCs to iChondrocytes. **b** Morphological and histopathological evaluation of iChondrocyte pellets at day 43, visualized by Alcian blue, safranin O, and toluidine blue staining (n = 6). Scale bar:100 um. **c** Relative mRNA expression levels of pluripotency markers (*SOX2* and *OCT4*) in iPSCs, iMSCs and iChondrocytes (n = 6). **d** Relative mRNA expression levels of chondrocyte-specific and hypertrophic chondrocyte-associated marker genes in iMSCs and iChondrocytes (n = 6). **e** Representative TEM images of sEVs (n = 3). Scale bar:100 nm. **f** Western Blot analysis of sEVs--specific marker (CD9, CD63, and Alix) (n = 3). **g** Size distribution analysis of sEVs, as determined by NTA (n = 3). Data are presented as mean ± SD. ∗*P* < 0.05, ∗∗*P* < 0.01, ∗∗∗*P* < 0.001.

Following the condensation into micro-pellets and subsequent chondrogenic differentiation from iMSCs, iChondrocytes were harvested between days 36 and 50. The detailed differentiation protocol is illustrated in Fig. 1a. Histochemical staining with Alcian blue, safranin O, and toluidine blue revealed strong positivity in iChondrocytes, indicative of substantial ECM deposition (Fig. 1b). Notably, the expression of pluripotency markers *SOX2* and *OCT4* was undetectable in iChondrocytes (Fig. 1c), while the expression levels of mature chondrocyte markers *ACAN*, *COL2A1*, and *SOX9* peaked between days 36 and 43 (Fig. 1d). Given the progressive increase in the expression of hypertrophic chondrocyte markers *COL10A1*, *IHH*, and *MMP13*, which reached peak levels by day 50 (Fig. 1d), iChondrocyte-derived small extracellular vesicles (iChondrocyte-sEVs) collected between days 36 and 43 were selected as the source material for subsequent experiments (the concentration of the sEVs original solution was 5.70 × 10^10^, and the protein concentration was 0.34 ug/uL). Scanning electron microscopy (SEM) analysis confirmed that sEVs derived from iPSCs, iMSCs, and iChondrocytes all exhibited the typical sEVs morphology (Fig. 1e). Furthermore, all three sEVs groups expressed the canonical sEVs markers CD9, CD63, and Alix (Fig. 1f). Nanoparticle tracking analysis (NTA) revealed that iPSC-sEVs, iMSC-sEVs, and iChondrocyte-sEVs had average particle sizes of 114.2 nm, 139.9 nm, and 136.4 nm, respectively (Fig. 1g).

### 3.2 IChondrocyte-sEVs promoted chondrogenesis

**Fig. 2:**
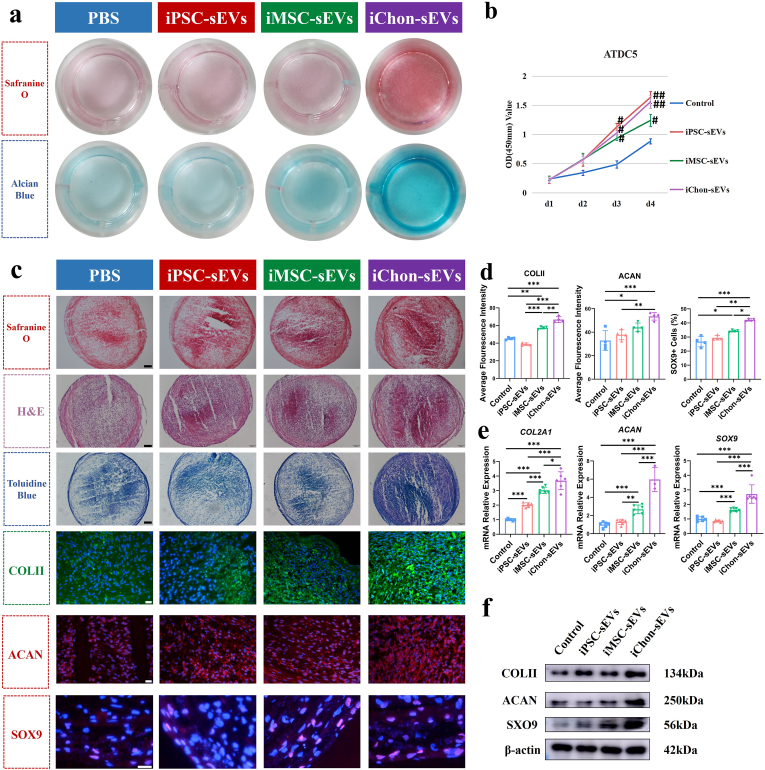
IChondrocyte-sEVs promoted chondrogenesis. **a** Representative image of safranin O and Alcian blue staining of ATDC5 cells after 14 days of chondrogenesis induction in a 24-well plate (n = 5). **b** Proliferation curves of ATDC5 cells were monitored from day 1 to day 4 (n = 5). **c** Representative images of histopathological and immunofluorescence staining of MSCs following 21 days of chondrogenesis induction (n = 4). Scale bars: black = 100 um, white = 20 μm **d** Quantitative analysis of the average fluorescence intensity of COLII, ACAN and SOX9 based on the results shown in (**c**) (n = 4). **e** Relative mRNA expression levels of anabolic genes in MSCs after 21 days of chondrogenesis induction (n = 6). **f** Western blotting analysis of COLII, ACAN and SOX9 in MSCs after 21 days of chondrogenesis induction (n = 4). Data are presented as mean ± SD. *∗P* < 0.05, ∗∗*P* < 0.01, ∗∗∗*P* < 0.001; ^#^*P* < 0.05, ^##^*P* < 0.01 vs. control group.

The ATDC5 cell line is a well-established murine chondrogenic cell line, widely used as a model for studying chondrocyte differentiation. In the differentiation assays utilizing ATDC5 cells, neither iPSC-sEVs nor iMSC-sEVs exhibited the capacity to induce spontaneous differentiation of ATDC5 cells. In contrast, iChondrocyte-sEVs demonstrated a marked enhancement in ATDC5 cell differentiation, as evidenced by intensified staining with both safranin O and alcian blue (Fig. 2a). Moreover, our investigations revealed that all sEVs subtypes facilitated the proliferation of ATDC5 cells. Notably, iChondrocyte-sEVs exhibited a pronounced proliferative effect on ATDC5 cells, with efficacy comparable to that of iPSC-sEVs (Fig. 2b). While the robust proliferative capacity of iPSC-sEVs may be intrinsically linked to the inherent rapid proliferation potential of iPSCs, the enhanced proliferative effect observed with iChondrocyte-sEVs is more plausibly attributed to their cellular affinity with ATDC5 cells and the presence of specific bioactive components within these vesicles.

To investigate the effect of sEVs on chondrogenesis, we induced the differentiation of MSCs into chondrocytes. We observed that iChondrocyte-sEVs significantly enhanced ECM deposition, as quantitatively demonstrated by intensified safranin O/HE/toluidine blue staining (Fig. 2c). Immunofluorescence analysis revealed that iChondrocyte-sEVs treatment markedly improved the chondrogenic differentiation capacity of MSCs, as evidenced by upregulated expression of characteristic chondrocyte markers, including COLII and ACAN. Moreover, there was an improvement in the structural organization of the ECM network within chondrocyte pellets following iChondrocyte-sEVs treatment. These findings were supported by statistical results on fluorescence intensity (Fig. 2d), along with a notable increase in the proportion of SOX9-positive cells in iChondrocyte-sEVs treatment group (Fig. 2c and d). Molecular analysis revealed that iChondrocyte-sEVs treatment significantly upregulated the expression of key chondrogenic markers at the transcriptional and protein level, including ACAN, COLII, and SOX9 (Fig. 2e and f). These collective findings provide compelling evidence that iChondrocyte-sEVs exert a potent stimulatory effect on chondrogenesis, enhancing both the molecular and structural characteristics of chondrogenic differentiation.

### 3.3 IChondrocyte-sEVs demonstrated significant regulatory effects on maintaining the homeostasis of chondrocyte-pellets

**Fig. 3:**
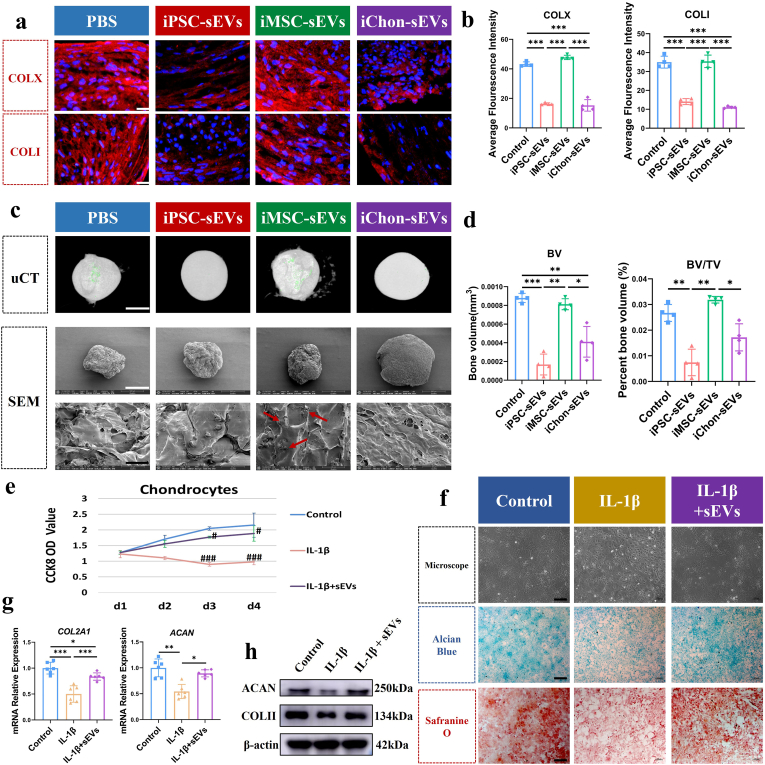
IChondrocyte-sEVs inhibited fibrosis and calcification in MSC-derived chondrocyte-pellets, as well as ameliorated impairment in OA chondrocytes. **a** Representative immunofluorescence images of MSC-derived chondrocytes pellets (n = 4). Scale bar: 20 μm **b** Quantitative analysis of mean fluorescence intensity for COLX and COLI across experimental groups (n = 4). **c** Microstructural characterization of MSC-derived chondrocyte pellets with μCT and SEM (n = 4). Calcified regions are indicated by green fluorescence, with calcium salt deposition marked by red arrows. Scale bar: white = 500 μm, black = 20 μm **d** Quantitative assessment of bone volume and bone volume percentage in MSC-derived chondrocytes pellets (n = 4). **e** OA chondrocytes proliferation was assessed over a 4-day period (n = 3). **f** Representative images illustrating cellular morphology, Safranin O staining, and Alcian blue staining of chondrocytes (n = 3). Scale bar:200 um. **g** Relative mRNA expression levels of *COL2A1* and *ACAN* genes in chondrocytes (n = 6). **h** Western blotting analysis of COLII and ACAN in chondrocytes (n = 4). Data are presented as mean ± SD. ∗*P* < 0.05, ∗∗*P* < 0.01, ∗∗∗*P* < 0.001; ^#^*P* < 0.05, ^###^*P* < 0.001 vs. control group.

Under normal physiological conditions, cartilage is expected to maintain its hyaline phenotype without exhibiting hypertrophic differentiation or fibrosis. Consequently, we investigated the expression of hypertrophy and fibrosis markers during chondrogenic differentiation of MSCs induced by sEVs. iChondrocyte-sEVs demonstrated a significant capacity to mitigate hypertrophy and fibrotic differentiation in MSC-induced chondrocyte pellets. This was evidenced by the downregulation of key markers associated with hypertrophic chondrocytes (COLX) and fibrochondrocytes (COLI) (Fig. 3a and b), indicating their potential to preserve the native chondrocytic phenotype. In contrast, iMSC-sEVs upregulated the expression of COLX and COLI in the chondrocyte pellets (Fig. 3a and b). To evaluate calcification within the chondrocyte pellets, micro-computed tomography (μCT) scanning was employed. The results revealed that iMSC-sEVs significantly enhanced calcification, as indicated by green fluorescence, whereas iChondrocyte-sEVs exerted an opposing effect (Fig. 3c). Additionally, iChondrocyte-sEVs reduced both bone volume (BV) and the bone volume to total volume ratio (BV/TV) within the pellets Fig. 3d). At the microscopic level, SEM analysis of dehydrated chondrocyte pellets revealed that iChondrocyte-sEVs stimulation led to pellets with a larger volume and smoother surface texture, indicative of enhanced production of a denser cartilage matrix. Conversely, iMSC-sEVs stimulation resulted in the presence of mineral deposits on the pellet surfaces, further corroborating their role in promoting calcification (Fig. 3c). These findings collectively suggest that iChondrocyte-sEVs play a crucial role in maintaining chondrocyte homeostasis and preventing pathological differentiation, while iMSC-sEVs may drive chondrocytes toward a hypertrophic and calcified state.

To further investigate the therapeutic potential of iChondrocyte-sEVs on pathological chondrocytes, we established an *in vitro* OA chondrocyte model using interleukin-1β (IL-1β) stimulation. The OA chondrocytes were subsequently treated with iChondrocyte-sEVs to evaluate their regenerative effects. Our findings demonstrate that iChondrocyte-sEVs significantly enhanced the proliferative capacity of OA chondrocytes (Fig. 3e). Moreover, the treatment effectively restored normal cellular morphology and promoted ECM synthesis, as evidenced by histological analyses (Fig. 3f) and key matrix markers at the transcriptional and protein level (Fig. 3g and h).

On the other hand, given the multidirectional differentiation potential of MSCs, their differentiation into osteoblasts within the joint cavity microenvironment could adversely affect the maintenance of hyaline cartilage properties, which would be highly detrimental to cartilage repair. To further explore this, we investigated whether different groups of sEVs influence the osteogenic differentiation of MSCs. MSCs were cultured in an osteogenic induction medium and treated with various sEVs. Alizarin red and ALP staining revealed that iMSC-sEVs promoted the formation of calcium nodules and increased ALP production (Fig. S2a and b), along with enhanced expression of osteogenesis-related genes (Fig. S2c). Conversely, iChondrocyte-sEVs inhibited the production of calcium nodules and ALP (Fig. S2a and b), suggesting that iChondrocyte-sEVs impeded the osteogenic differentiation of MSCs.

It is worth noting that angiogenesis is strongly discouraged in order to maintain normal physiological homeostasis in articular cartilage. Therefore, we also examined the impact of sEVs on human umbilical vein endothelial cells (HUVECs, a common tool for angiogenesis experiments). Proliferation assays revealed that both iPSC-sEVs and iMSC-sEVs significantly enhanced the growth of HUVECs, whereas iChondrocyte-sEVs exhibited no observable effect (Fig. S3a). In scratch assays, iMSC-sEVs demonstrated robust pro-migratory activity, as evidenced by a marked reduction in scratch width within just 7 h, indicating superior migration-promoting capabilities compared to other groups. In contrast, iChondrocyte-sEVs suppressed HUVECs migration (Fig. S3b and c). The tube formation assay, a widely utilized *in vitro* model for assessing angiogenesis, revealed that iChondrocyte-sEVs significantly inhibited HUVECs tube formation. Specifically, iChondrocyte-sEVs reduced the number of tubules, nodes, master junctions, and total tube length compared to the iMSC-sEVs group (Fig. S3d and e), suggesting their potential to attenuate angiogenic processes. Given that vascularization of articular cartilage is physiologically undesirable under normal conditions, we propose that iChondrocyte-sEVs may play a role in maintaining cartilage homeostasis by inhibiting vascularization.

### 3.4 IChondrocyte-sEVs significantly enhanced cartilage regeneration in a murine model of OA

**Fig. 4:**
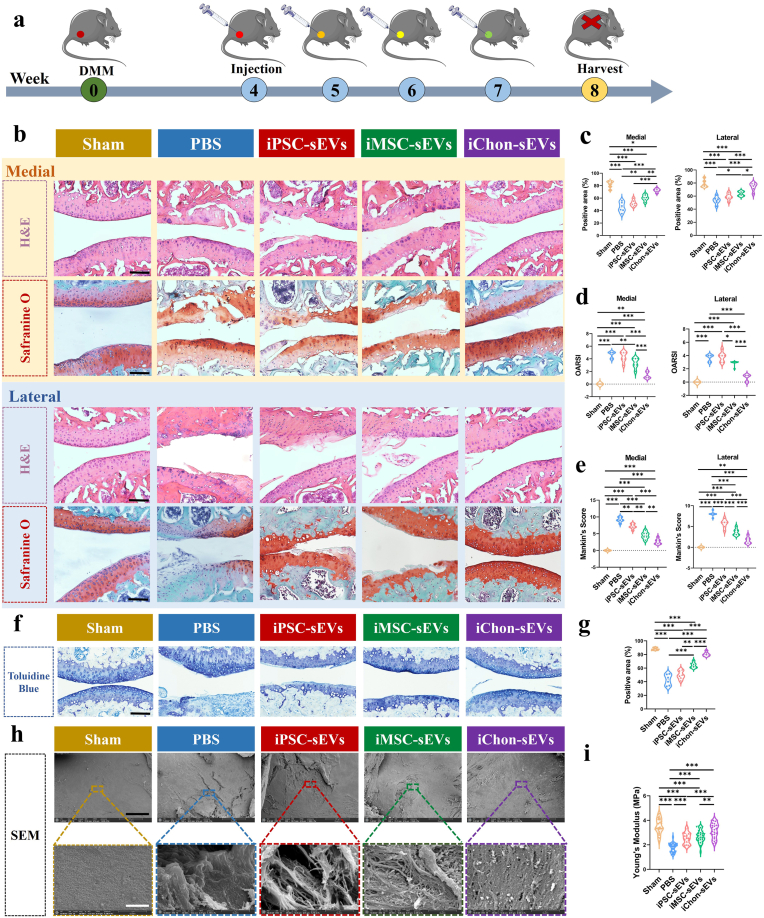
IChondrocyte-sEVs promoted cartilage repair in OA mice. **a** Schematic diagram illustrating the establishment of the OA mouse model and the administration of sEVs injections (n = 6). All groups except the sham group were conducted under DMM conditions. **b** Representative histopathological images of HE and Safranine O staining of the medial and lateral articular cartilage (n = 6). Scale bar: 200 μm. **c** Quantitative analysis of HE staining. **d-e** OARSI and Mankin's score of medial and lateral articular cartilage (n = 6). **f-g** Representative images and quantitative analysis of Toluidine Blue staining of the articular cartilage (n = 6). Scale bar: 200 μm **h** Representative SEM images of the articular cartilage surface morphology (n = 6). Scale bar: black = 100 um., white = 2 μm. **i** Quantitative analysis of Young's modulus of articular cartilage across experimental groups (n = 6). ∗*P* < 0.05, ∗∗*P* < 0.01, ∗∗∗*P* < 0.001.

Following the successful establishment of the OA mouse model, therapeutic administration of sEVs was performed as illustrated in Fig. 4a. To assess the potential toxicity of sEVs, we conducted histological staining of mouse visceral organs, which demonstrated the absence of *in vivo* toxicity (Fig. S4). To further investigate the retention capacity within the knee joint cavity and evaluate the penetration efficiency through the articular cartilage layer, we administered three distinct groups of fluorescently labeled sEVs into the murine joint cavity. Longitudinal observation demonstrated sustained retention of all sEVs groups within the joint cavity for over one week (Fig. S5a), with iChondrocyte-sEVs exhibiting the most pronounced retention efficacy (Fig. S5b). Subsequent analysis confirmed successful chondrocyte uptake of all three sEVs groups following their penetration through the cartilaginous matrix (Fig. S5c and d). Similarly, these sEVs were also taken up by synovial tissue (Fig. S5d).

Histopathological examination using HE and Safranin O staining revealed distinct phenotypic differences between the medial and lateral joint compartments following DMM surgery. We identified two characteristic phenotypes: a cartilage defect phenotype in the medial compartment and a fibrocartilaginous phenotype in the lateral compartment, both of which have been underreported in previous studies. While iPSC-sEVs and iMSC-sEVs showed limited capacity to restore medial cartilage fragmentation, iChondrocyte-sEVs demonstrated superior reparative potential, effectively restoring cartilage integrity. In the lateral compartment, iPSC-sEVs and iMSC-sEVs exhibited minimal impact on the degenerated cartilage layer, as evidenced by reduced Safranin O staining. In contrast, iChondrocyte-sEVs effectively promoted hyaline cartilage regeneration, characterized by matrix deposition and cartilage layer thickening (Fig. 4b). The quantitative analysis using HE staining also confirmed this (Fig. 4c). The comprehensive therapeutic efficacy of iChondrocyte-sEVs across both phenotypes underscores its remarkable regenerative potential. Furthermore, iChondrocyte-sEVs were found to significantly reduce the OARSI and Mankin's scores in both the medial and lateral joints. However, this effect was not observed with iPSC-sEVs and iMSC-sEVs (Fig. 4d and e).

Toluidine blue staining revealed significant loss of glycosaminoglycan (GAG) expression in the cartilage matrix post-DMM surgery, which was effectively restored by iChondrocyte-sEVs treatment (Fig. 4f and g). SEM analysis demonstrated substantial microstructural damage following DMM surgery, including surface fissures and disorganized collagen fibers. However, iChondrocyte-sEVs treatment resulted in improved cartilage microstructure with reduced collagen damage (Fig. 4h).

To further evaluate cartilage health, we assessed mechanical properties through nanoindentation analysis. Measurement of the Young's modulus revealed that all three sEVs groups contributed to the restoration of cartilage mechanical strength. Notably, the iChondrocyte-sEVs group demonstrated the highest Young's modulus values, indicating superior cartilage repair efficacy (Fig. 4i).

### 3.5 IChondrocyte-sEVs ameliorated the multifaceted pathological phenotypes observed in the DMM-induced OA murine model

**Fig. 5:**
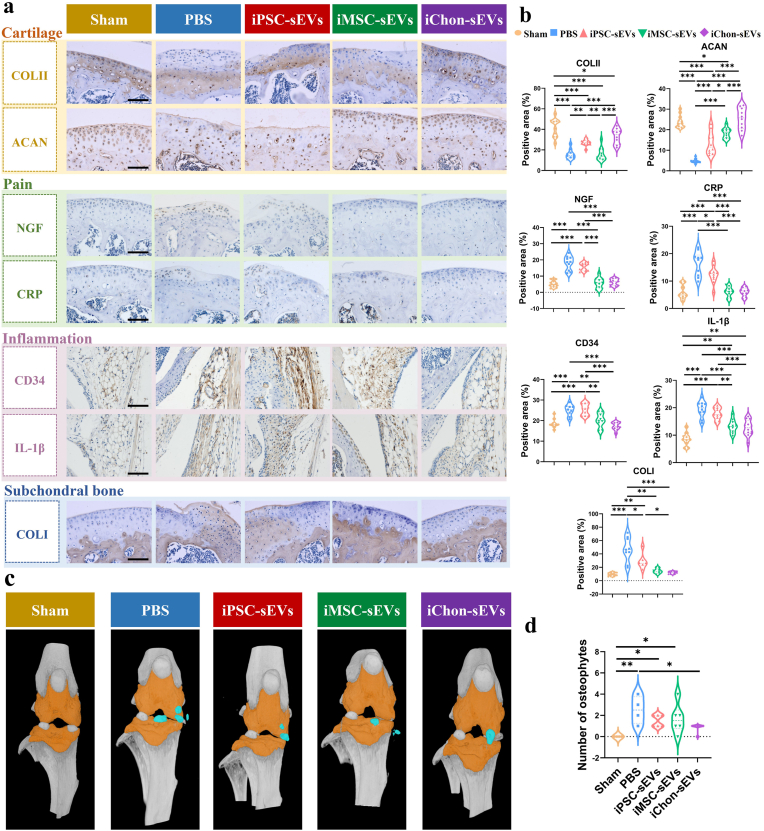
IChondrocyte-sEVs alleviated multiple OA phenotypes. **a** Representative histopathological images demonstrating key protein markers associated with the four characteristic phenotypes of OA (n = 6). Scale bar: 200 μm **b** Semi-quantitative analysis of histopathological features shown in (**a**). **c** Representative uCT imaging of murine joints. The three-dimensional reconstruction shows subchondral bone (yellow) and osteophytes (blue) (n = 6). **d** Quantitative analysis of osteophytes (n = 6). ∗*P* < 0.05, ∗∗*P* < 0.01, ∗∗∗*P* < 0.001. All groups except the sham group were conducted under DMM conditions.

Given that OA is a heterogeneous disease with various clinical phenotypes [22] and impacts the whole joint. Previous studies on OA in murine models have rarely addressed the phenotypic classification of OA. Therefore, this study aimed to investigate whether consistent OA phenotypes would manifest in mice following DMM surgery and to determine which phenotypes could be ameliorated by sEVs treatment. To achieve these objectives, we utilized well-characterized marker proteins specific to each phenotype. Our results demonstrated that iChondrocyte-sEVs partially restored the physiological expression of critical cartilage markers—COLII and ACAN—within the cartilage layer, specifically addressing the cartilage phenotype (Fig. 5a). Following DMM surgery, nerve growth factor (NGF) and C-reactive protein (CRP) proteins were prominently expressed within the cartilage layer; however, administration of iChondrocyte-sEVs markedly attenuated their expression levels (Fig. 5a). Within the inflammatory phenotype, surgical induction of DMM resulted in a marked upregulation of synovial expression of the inflammatory mediators CD34 and IL-1β. Notably, administration of iChondrocyte-sEVs exhibited a significant capacity to attenuate the expression levels of these inflammatory markers in the synovial tissue (Fig. 5a). These findings were further corroborated by quantitative statistical analysis of positively stained regions for each respective marker protein (Fig. 5b).

In the subchondral bone phenotype analysis, ectopic expression of COLI was observed in the cartilage layer following DMM surgery, whereas iChondrocyte-sEVs treatment significantly suppressed COLI expression in the same region (Fig. 5a and b). Osteophytes, a hallmark feature of OA progression, were further evaluated as part of this study. Micro-CT imaging of mouse joints revealed distinct structural changes, with the rendered yellow regions representing the subchondral bone and the blue areas indicating sites of osteophyte formation (Fig. 5c). Quantitative analysis demonstrated a marked increase in osteophyte formation post-DMM surgery; however, this effect was attenuated in the iChondrocyte-sEVs treatment group, which exhibited significantly fewer osteophytes (Fig. 5d). These findings suggest that iChondrocyte-sEVs exert inhibitory effects on OA-associated osteophyte formation. Collectively, the data indicate that iChondrocyte-sEVs mitigate key pathological features of OA, underscoring their multifaceted therapeutic potential in cartilage repair and disease modification.

Furthermore, iChondrocyte-sEVs have been demonstrated to attenuate degenerative changes in the meniscus of OA mice, enhance the expression of COLII, and reduce the levels of inflammatory markers (CD34, IL-1β) as well as pain-associated factors (NGF, CRP) in the meniscus (Fig. S6). These findings further substantiate the therapeutic potential of iChondrocyte-sEVs in the management of OA.

### 3.6 IChondrocyte-sEVs improved joint mobility and improved motor function in OA murine mode

**Fig. 6:**
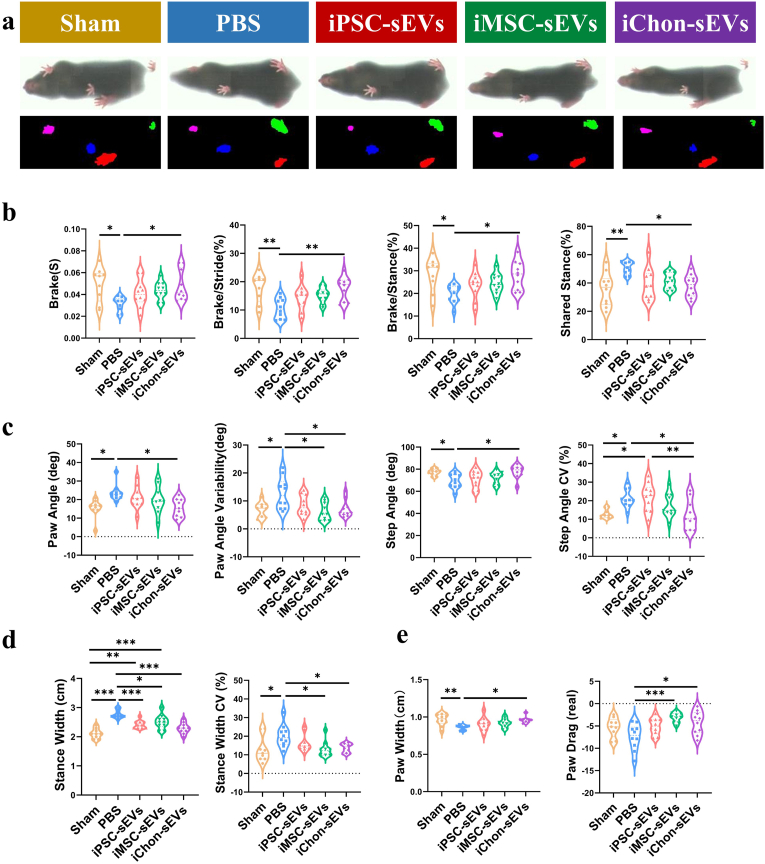
IChondrocyte-sEVs improved joint motor function in OA mice. **a** Schematic representation of gait analysis in mice, utilizing a four-color fluorescence system to depict limb footprints (n = 10). **b** Statistical analysis of temporal parameters in hind limb gait (n = 10). **c** Statistical analysis of paw contact angles during hind limb gait (n = 10). **d** Statistical analysis of stance width in hind limb gait (n = 10). **e** Statistical analysis of paw contact area during hind limb gait (n = 10). ∗*P* < 0.05, ∗∗*P* < 0.01, ∗∗∗*P* < 0.001. All groups except the sham group were conducted under DMM conditions.

Given the profound impact of OA on joint mobility, the primary therapeutic objective should be centered on functional improvement. To this end, the DigiGait™ imaging system was employed to conduct gait analysis across all murine groups, aiming to assess the potential of sEVs in enhancing motor function in murine joints. Fig. 6a provides a schematic illustration of the gait detection process in mice.

In temporal analysis, iChondrocyte-sEVs significantly prolonged brake time and its proportion (Fig. 6b). The “brake” phase, defined as the period from initial paw contact to maximum paw contact—may reflect more precise load distribution and reduced peak loads when prolonged. The observed increase in brake time and its proportion suggests that iChondrocyte-sEVs treatment enhances the precision of hind limb control. Concurrently, iChondrocyte-sEVs significantly reduced the shared stance duration of the hind limbs, indicating improved strength and control.

A comparative analysis of paw contact angles revealed that iChondrocyte-sEVs administration significantly ameliorated paw angles while concurrently enhancing step angles (Fig. 6c). The paw angle, clinically referred to as external rotation, typically maintains a normative value of approximately 15° in the hind paw of healthy subjects. Pathological elevation of this parameter has been consistently correlated with the development of motor system disorders. The step angle, defined as the angular displacement between the left and right hind paws, is biomechanically determined by stride length and stance width parameters. In the present experimental model, OA mice demonstrated characteristic pathological alterations, manifesting as increased paw angles and reduced step angles. The therapeutic intervention with iChondrocyte-sEVs demonstrated significant modulatory effects on these parameters, suggesting potential restoration of hindlimb motor functionality.

Stance width, defined as the mediolateral distance between the center points of the bilateral hind paws during static standing, serves as a critical parameter for postural stability assessment. Increased stance width typically reflects compensatory postural adjustments to maintain body equilibrium. Notably, intra-articular administration of iChondrocyte-sEVs demonstrated significant therapeutic effects, as evidenced by the reduction in both stance width and its coefficient of variation (CV), suggesting improved locomotor stability (Fig. 6d). Furthermore, quantitative analysis of paw contact patterns revealed that iChondrocyte-sEVs treatment effectively restored normal paw width parameters and markedly reduced pathological paw dragging behavior, indicating substantial improvement in gait parameters (Fig. 6e). Among all the interventions evaluated, iChondrocyte-sEVs demonstrated the most pronounced enhancement in motor function in murine models, attributable to their superior functional characteristics.

### 3.7 Proteomic profiling of iChondrocyte-sEVs revealed their distinctive functional characteristics and molecular signatures

**Fig. 7:**
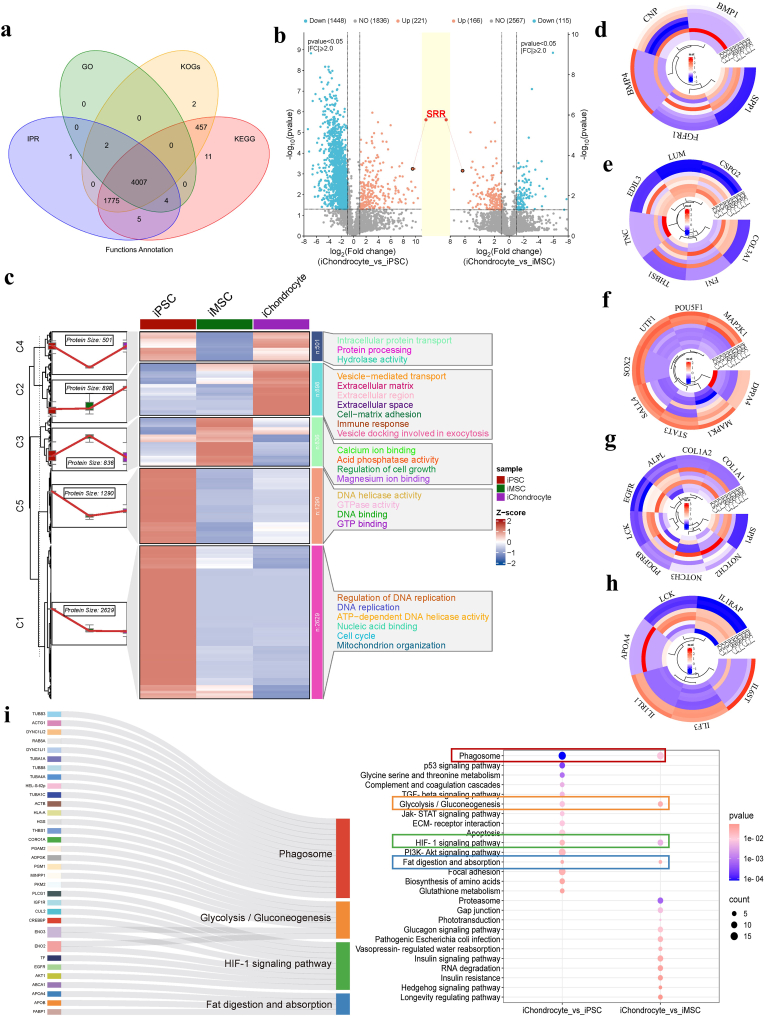
Proteomic profiling of sEVs. **a** Venn diagram depicting the functional annotation of proteins derived from GO, KEGG, KOG, and IPR databases. **b** Volcano plot illustrating differentially expressed proteins in sEVs proteome (n = 3). **c** GO enrichment analysis of sEVs proteome (n = 3). **d** Circular cluster analysis of chondrogenesis-associated proteins across three sEVs groups (n = 3). **e** Circular cluster analysis of extracellular matrix-related proteins across three sEVs groups (n = 3). **f** Circular cluster analysis of pluripotency-associated proteins across three sEVs groups (n = 3). **g** Circular cluster analysis of calcification- and vascularization-related proteins across three sEVs groups (n = 3). **h** Circular cluster analysis of inflammation-associated proteins across three sEVs groups (n = 3). **i** KEGG pathway enrichment analysis of sEVs proteome (n = 3).

Given that the functional properties of sEVs are predominantly governed by their molecular cargo, a thorough characterization of their internal constituents is essential for elucidating the mechanistic basis of iChondrocyte-sEVs. To this end, we implemented the 4D-label-free proteomic platform to quantitatively assess the relative expression profiles of the entire proteome within sEVs. Our proteomic analysis successfully identified more than 6000 distinct proteins within the sEVs proteome (Fig. 7a).

A comparative proteomic analysis was performed to identify differentially expressed proteins among iChondrocyte-sEVs, iPSC-sEVs, and iMSC-sEVs populations. The volcano plot analysis demonstrated substantial proteomic differences between iChondrocyte-sEVs and iPSC-sEVs, with 221 proteins showing significant up-regulation and 1448 proteins exhibiting down-regulation. When compared to iMSC-sEVs, iChondrocyte-sEVs displayed 166 up-regulated and 115 down-regulated proteins (fold change >2, P < 0.05). These distinct protein expression patterns provide compelling evidence for the successful induction and differentiation of iPSCs into chondrocyte lineage cells (Fig. 7b).

The Gene Ontology (GO) enrichment analysis of the proteomes from the three sEVs groups (Fig. 7c) demonstrated segregation into five distinct clusters, each displaying unique expression patterns across the groups. Notably, cluster 2 exhibited significantly higher expression levels exclusively in iChondrocyte-sEVs. The proteins within this cluster are primarily associated with ECM, immune response, vesicle-mediated transport, and related biological processes. These findings underscore the functional role of iChondrocyte-sEVs in enhancing ECM production and mitigating inflammation, as observed in *in vivo* experiments. In contrast, cluster 3 showed exclusive high expression levels in iMSC-sEVs. The proteins in this cluster are predominantly involved in molecular functions such as calcium ion binding, acid phosphatase activity, and regulation of cell growth. These functions may underlie the observed promotion of cell proliferation and cartilage calcification in cytological experiments involving iMSC-sEVs. Additionally, iPSC-sEVs demonstrated elevated expression levels in cluster 1, which encompasses biological processes such as DNA replication and cell cycle as well as cluster5 comprising molecular functions such as DNA binding and DNA helicase activity. This suggests that the primary role of iPSC-sEVs may remain centered on supporting cell proliferation and maintaining stemness characteristics, with limited contribution to cartilage regeneration.

Based on the distinct functional profiles observed in our *in vitro* and *in vivo* experiments across the three groups of sEVs, we sought to identify differentially expressed proteins that could provide mechanistic explanations for these experimental findings. Proteins associated with diverse biological processes in the three sEVs groups were systematically clustered and visualized using circular cluster analysis. The clustering analysis revealed that iChondrocyte-sEVs exhibited upregulated expression of proteins related to cartilage development, alongside downregulated expression of proteins associated with hypertrophy promotion (Fig. 7d). This pattern suggests a potential role for iChondrocyte-sEVs in promoting chondrogenesis. Furthermore, iChondrocyte-sEVs displayed elevated expression levels of matrix-related proteins, indicative of enhanced ECM synthesis (Fig. 7e). These findings offer mechanistic insights into the capacity of iChondrocyte-sEVs to facilitate cartilage regeneration in both *in vitro* and *in vivo* settings. Of particular note, iChondrocyte-sEVs demonstrated a marked reduction in the expression of pluripotency-related proteins (Fig. 7f), suggesting a lower risk of carcinogenicity and an improved safety profile. Furthermore, iChondrocyte-sEVs exhibited low expression levels of calcification- and vascularization-related proteins [23] compared to iMSC-sEVs (Fig. 7g), which aligned with cytologic assay results. Additionally, iChondrocyte-sEVs displayed decreased expression of pro-inflammatory proteins [24] (Fig. 7h), suggesting a significant inhibitory effect on inflammation, which is consistent with previous experimental observations regarding the inflammatory phenotype in murine models of OA.

To elucidate the molecular mechanisms underlying the functional properties of iChondrocyte-sEVs, we performed Kyoto Encyclopedia of Genes and Genomes (KEGG) pathway enrichment analysis on differentially expressed proteins identified within iChondrocyte-sEVs. Proteomic profiling revealed significant enrichment and upregulation of proteins associated with four key signaling pathways: HIF-1 signaling, Phagosome, Glycolysis/Gluconeogenesis, and Fat digestion and absorption (Fig. 7i). Notably, the HIF-1 and Phagosome pathways were identified as critical regulators of chondrocyte fate determination, while the Glycolysis/Gluconeogenesis and Fat digestion and absorption pathways were found to be predominantly involved in metabolic regulation of chondrocytes. These findings collectively demonstrate that these four interconnected pathways play pivotal roles in modulating chondrocyte function and homeostasis.

### 3.8 The modulation of P38/ERK signaling pathway by SRR may constitute a significant molecular mechanism underlying the function properties of iChondrocyte-sEVs

**Fig. 8:**
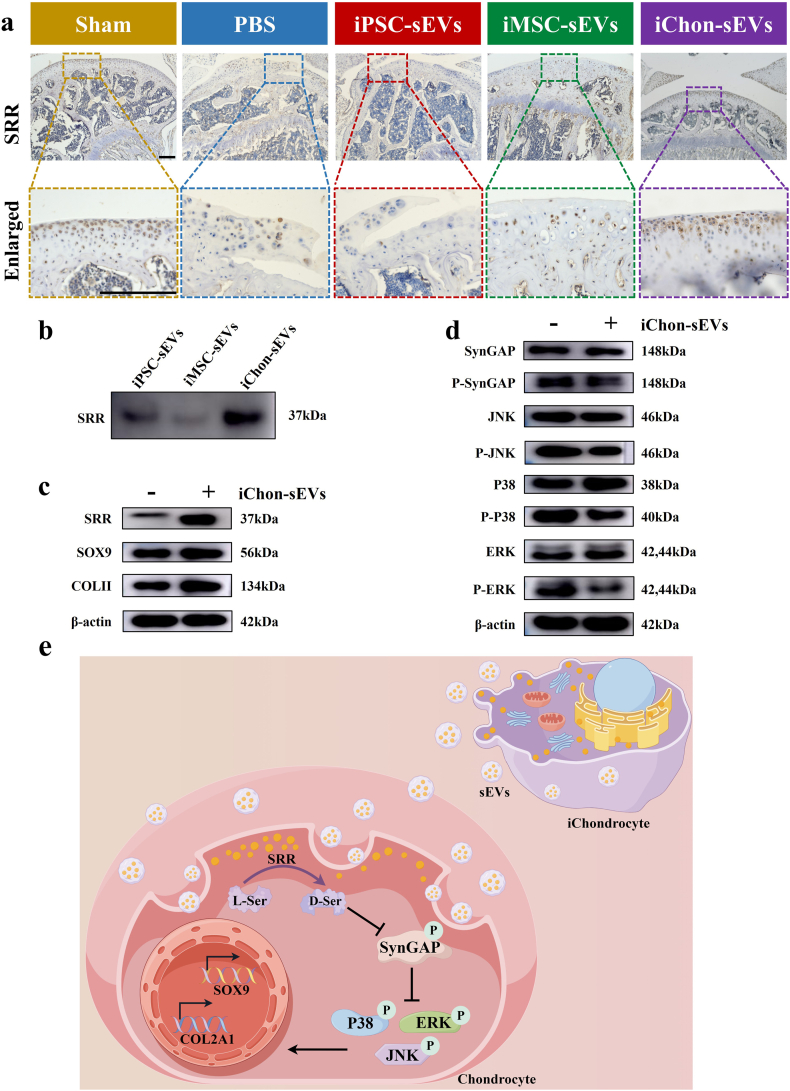
iChondrocyte-sEVs-derived SRR facilitated cartilage regeneration via the P38/ERK pathway. **a** Representative histopathological image of SRR in articular cartilage (n = 6). Scale bar: 200 μm **b** Western Blot analysis of SRR levels in iPSC-sEVs, iMSC-sEVs and iChondrocyte-sEVs (n = 3). **c** Western Blot analysis of SRR, SOX9, COLII expression in MSC-derived chondrocytes treated with or without iChondrocyte-sEVs (n = 3). **d** Western Blot analysis of key proteins in the P38/ERK signaling pathway in MSC-derived chondrocytes treated with or without iChondrocyte-sEVs (n = 3). **e** Schematic diagram illustrating the molecular mechanism by which iChondrocyte-sEVs exert their regenerative effects. All groups except the sham group were conducted under DMM conditions.

Our proteomic screening revealed that SRR was specifically and highly expressed in the iChondrocyte-sEVs (Fig. 7b). This finding was subsequently validated through Western blot analysis (Fig. 8b). To further corroborate the expression pattern of SRR, we conducted comprehensive histopathological staining, which demonstrated predominant SRR expression in the superficial zone of normal articular cartilage. Notably, following DMM surgery, a marked reduction in SRR expression was observed. Intriguingly, administration of iChondrocyte-sEVs significantly restored SRR expression in the cartilage layer (Fig. 8a, tibial side of the articular cartilage). Previously, SRR mRNA was detected in the chondrocyte layer of P1 rat tibia [25], and our findings further corroborated the expression of SRR protein in the adult mouse cartilage layer. Previous studies have established that SRR catalyzes the synthesis of D-serine from L-serine, a key co-agonist for N-methyl-D-aspartate (NMDA) receptors. NMDA receptors, which function as calcium channels, mediate increased calcium influx upon activation. This process plays a critical role in the mechanical induction of articular chondrocytes and the maintenance of ECM homeostasis in joints. We hypothesized that iChondrocyte-sEVs promote chondrogenic differentiation by enhancing intracellular D-serine levels through the delivery of SRR protein. Elevated D-serine levels activate NMDA receptors, leading to increased calcium influx. This, in turn, reduces the levels of phosphorylated synaptic GTPase-activating protein (P-SynGAP), thereby inhibiting the activity of the P38/ERK signaling pathway.

To investigate the specific mechanism by which iChondrocyte-sEVs deliver SRR to treat OA cartilage, we examined the effects of iChondrocyte-sEVs during chondrogenic induction of MSCs. We observed that iChondrocyte-sEVs increased cellular SRR protein levels and upregulated the expression of cartilage matrix markers, including SOX9 and COLII (Fig. 8c). Concurrently, the expression levels of P-SynGAP, P-JNK, P-P38, and P-ERK were significantly reduced following iChondrocyte-sEVs treatment (Fig. 8d). These findings suggest that SRR delivered by iChondrocyte-sEVs promotes chondrogenic differentiation by suppressing the activity of the P38/ERK signaling pathway (Fig. 8e).

## 4 Discussion

For biological applications, sEVs possess a multitude of advantages [26], including non-toxicity and immune compatibility, targeted localization to injured cells [27] and tissue [28], direct delivery of their contents across cellular membranes and the blood–brain barrier [29], age-independent efficacy and maintenance of biological characteristics after long-term storage. For cell-free therapy of cartilage injury, the permeability of conventional small molecule drug therapies is limited by the dense cartilage matrix, whereas sEVs can effectively penetrate the cartilage layer to deliver their contents [30]. Although natural chondrocyte-sEVs have demonstrated the ability to enhance the proliferation and differentiation of cartilage progenitor cells, as well as inhibit angiogenesis [11], their utilization is limited due to the scarcity and limited proliferative capacity. To overcome this limitation, we utilized iPSCs possessing unlimited proliferative capacity as a valuable source of chondrocytes, and their chondrogenic differentiation significantly augmented the production of iChondrocyte-sEVs. Furthermore, future comparative studies with sEVs derived from primary chondrocytes will be valuable to determine if the iPSC-derived source offers functional advantages, such as superior consistency, scalability, or a more ‘rejuvenated’ cargo profile devoid of donor-related variations or age-related senescence.

A key strength of our approach lies in the use of a 3D pellet culture system to generate iChondrocyte-sEVs. This method better recapitulates the native chondrogenic microenvironment than 2D monolayer culture, potentially yielding sEVs with a cargo enriched for pro-chondrogenic functions. We posit that these 3D-derived sEVs offer superior therapeutic advantages by more effectively promoting ECM synthesis and exerting enhanced anti-hypertrophic, anti-calcification, and anti-angiogenic activities, which are critical for cartilage regeneration and homeostasis, compared to sEVs from dedifferentiated 2D-cultured chondrocytes [31]. Therefore, the utilization of 3D-cultured iChondrocyte-sEVs in our therapeutic strategy is not merely a technical choice but a fundamental aspect that likely underpins their enhanced efficacy in promoting cartilage regeneration and homeostasis, as observed in our experimental results.

Previous studies have initially demonstrated that chondrocyte-sEVs possessed the ability to enhance the chondrogenic differentiation and deposition of ECM in MSCs [32,33]. Our study further confirmed same functionality of iChondrocyte-sEVs, aligning with previous findings. Furthermore, although previous studies have demonstrated some beneficial effects of MSC-sEVs on chondrocytes [34], researchers have attempted to compare the functional disparities between Chondrocyte-sEVs and MSC-sEVs. It was observed that MSC-sEVs facilitated hypertrophic differentiation of engineered cartilage constructs accompanied by vascular ingrowth, whereas Chondrocyte-sEVs promoted proliferation and chondrogenic differentiation of these cartilage constructs while inhibiting angiogenesis, exhibiting a more remarkable capacity. Our results not only aligned with these findings but also offered a comprehensive and multi-dimensional perspective by revealing the capacity of iChondrocyte-sEVs to maintain chondrocyte homeostasis. As the function of sEVs depends on the cells from which they are derived, these experimental results provided additional evidence supporting the remarkable similarity between iChondrocyte and native chondrocytes.

For a considerable period, one of the most concerning issues regarding iPSCs and their derivatives has been the high risk of tumor formation [35] due to their robust proliferative capacity. Although we have successfully differentiated iPSCs into iChondrocytes through an extensive induction program that greatly diminishes pluripotency gene expression, it remains uncertain whether undifferentiated iPSCs persist within the iChondrocyte population. The substantial decrease in pluripotency proteins observed in iChondrocyte-sEVs suggested a significant reduction in tumorigenicity when utilizing these sEVs, thereby providing assurance for their safe application.

KEGG enrichment analysis was conducted on the proteome data of sEVs to elucidate the underlying mechanism by which iChondrocyte-sEVs regulate cartilage regeneration. All four signaling pathways exert significant effects on chondrocytes function. The impact of the HIF-1 pathway on cartilage has been substantiated by a plethora of studies [36,37]. The HIF-1 pathway exerted its influence in various aspects, including the treatment of OA, degradation of cartilage ECM, apoptosis, inflammatory response, autophagy [38]. Our proteomic analysis has further elucidated that the HIF-1 signaling pathway plays a potentially crucial role in mediating the cartilage repair mechanisms of iChondrocyte-sEVs, thereby providing significant mechanistic insights that complement and extend previous findings in this field. Additionally, the association between Phagosomes and cartilage has been scarcely investigated. A previous study has examined the pivotal role of N-glycoproteomics in the pathological mechanisms underlying Kaschin-Beck disease and OA revealing initial evidence suggesting that N-glycosylation may regulate chondrocyte integrity through modulation of the Phagosome pathway [39]. Our KEGG enrichment analysis further corroborated that the Phagosome pathway may represent a pivotal mechanism underlying iChondrocyte-sEVs-mediated cartilage repair. The current association studies between Glycolysis/Gluconeogenesis and cartilage/chondrocytes primarily focuse on individual cases and superficially explores genetic differences, such as the genetic changes in the Glycolysis/Gluconeogenesis pathways during the process of chondrogenic differentiation of MSCs [40], in cartilage mechanical injury [41] or in OA patients [42]. For the first time, we have demonstrated that iChondrocyte-sEVs may restore the cartilage phenotype by modulating the Glycolysis/Gluconeogenesis pathways at the protein level. The dysregulation of cholesterol influx and efflux in chondrocytes could result in intracellular cholesterol accumulation, ultimately leading to altered chondrocyte quality and impacting OA development [43]. We supposed that iChondrocyte-sEVs could potentially ameliorate cartilage degeneration through the modulation of cholesterol metabolism in chondrocytes. Collectively, these findings demonstrate that iChondrocyte-sEVs exert multifaceted effects on chondrocyte biology. The cumulative evidence suggests that targeted modulation of these signaling pathways may constitute a promising therapeutic approach for cartilage repair and regeneration.

SRR catalyzes the conversion of L-serine to D-serine [44] and is distributed across multiple tissues and systems, such as central nervous system, placenta, cardiac tissue, and skeletal muscle. The mRNA expression of SRR was detected in rat cartilage tissue, the overexpression of SRR in ATDC5 cells resulted in a significant inhibition of ALP activity, as well as a notable decrease in the mRNA expression of *Col10A1* [25]. In chondrocytes infected with AdV-SRR, a significant decrease was seen in ALP activity, and sustained exposure to D-Serine significantly decreased ALP activity and Ca^2+^ contents in chondrocytes [45], suggesting SRR's potential involvement in inhibiting chondrocytes hypertrophy and mineralization. Notably, our findings demonstrated that iChondrocyte-sEVs significantly upregulated SRR expression in the cartilage layer following DMM surgery, effectively restoring the normal cartilage phenotype and mitigating degeneration, potentially through the inhibition of the P38/ERK signaling pathways. These results provide the first *in vivo* evidence of the therapeutic role of SRR protein in cartilage repair, thereby corroborating and extending previous *in vitro* studies that highlighted its beneficial effects on chondrocyte function. While our data suggest that sEVs-derived SRR may attenuate the P38/ERK signaling pathway to promote chondrogenic differentiation, we acknowledge this mechanistic insight is preliminary and represents a limitation of our study. This specific pathway might be one of several mechanisms contributing to the observed effects. These findings provide a starting point, and the complete elucidation of this link, alongside the overall functional role of SRR and the contribution of other factors like miRNAs, will be a major focus of future work.

While our study provides evidence for the therapeutic potential of iChondrocyte-sEVs in mitigating OA progression in a rodent model, several translational aspects warrant further discussion to pave the way for clinical application. First, the long-term outcomes of such a treatment remain an open question. Future investigations employing longer observation periods, preferably in large animal models with spontaneous OA, are essential to assess the durability of cartilage repair, the long-term stability of the regenerated tissue, and the potential for any late-onset adverse effects. Second, the optimal repeated administration regimen needs to be defined. The chronic progression of OA [46] likely demands a repeated administration strategy, the parameters of which must be guided by pharmacokinetic studies of sEVs in the joint and the disease stage. Encouragingly, our current data show that iChondrocyte-sEVs have promising joint retention and cartilage-targeting properties, which could support an extended dosing cycle. Defining the requisite number of injections and their intervals will thus be a pivotal step in translating this therapy into a clinical protocol. Finally, in this study, we isolated iChondrocyte-sEVs using differential ultracentrifugation, a widely accepted method in the research field. However, this method presents limitations for clinical-grade manufacturing, including its time-consuming nature, limited throughput, and challenges in maintaining a closed and sterile system. Therefore, addressing the scale-up feasibility represents a pivotal challenge for clinical translation. Translating our findings to the clinic will require the development of robust, scalable, and reproducible production processes under GMP conditions. This entails adopting readily scalable isolation strategies [47] —such as size-exclusion chromatography (SEC), which is particularly attractive for its ability to preserve EV bioactivity and enable standardized operation, or tangential flow filtration (TFF)—alongside the use of bioreactors for cell expansion and the establishment of stringent quality control measures to ensure batch-to-batch consistency. Consequently, while the present study provides fundamental insights, future work will focus on transitioning to these scalable, GMP-compliant production methods to ultimately enable clinical applications.

In summary, our study has successfully developed a robust system for isolating iChondrocyte-sEVs from iPSCs. For the first time, we have demonstrated the critical role of iChondrocyte-sEVs in regulating chondrogenesis, maintaining chondrocyte homeostasis, and promoting cartilage repair and regeneration. Through comprehensive proteomic profiling, we have further elucidated the molecular mechanisms underlying the functions of iChondrocyte-sEVs. While our study has certain limitations, including a relatively short observation period and incomplete characterization of specific effector molecules and pathways, our findings establish a novel cell-free therapeutic approach for cartilage regeneration. Importantly, the absence of pluripotency-related proteins in iChondrocyte-sEVs supports their safety profile for potential clinical applications.

## Ethics approval and consent to participate

All animal experimental procedures were approved by the Ethics in Experimental Animal Center of the Fourth Military Medical University. Title: Research on the Role and Mechanism of Extracellular Vesicles in Cartilage Repair. Approval number: IACUC-20230023. Date of approval: May 10th,2023.

## Availability of data and material

All data analyzed during this study are included in this published article and supplementary material.

## Authors’ contributions

Y.Q. Hu performed most of the experiments and wrote the manuscript; P.Z Cheng designed part of the experiments; M.G Han performed part of the *in vitro* experiments; L.Y Jia, X. Hao and C.X. Zhou performed part of the *in vivo* experiments; G.Y. Ding, J. Fan and W.G Lu provided technical and resource assistance throughout the project; Z.J. Luo and L. Yang revised the manuscript and provided funds. All authors have given approval to the final version of the manuscript.

## Declaration of AI and AI-assisted technologies in the writing process

The authors declare that they have not use AI-generated work in this manuscript.

## Funding

The project has been supported by 10.13039/501100001809National Natural Science Foundation of China (82394442, 82130070, 82002351), 10.13039/501100015401Key Research and Development Projects of Shaanxi Province (2022SF-331, 2024SF-YBXM-375) and Natural Science Basic Research Program of Shaanxi (2025JC-YBQN-1098).

## Conflicts of interest statement

The authors have declared that no competing interest exists.
