# Supplementary material for: Extracellular vesicles originating from induced pluripotent stem cell-derived chondrocytes facilitate the regeneration of osteoarthritic cartilage
Source: J Orthop Translat. 2026 Jan 10;56:101035. doi: 10.1016/j.jot.2025.101035 (PMC12988518; doi:10.1016/j.jot.2025.101035)
Supplement: Multimedia component 1 [file mmc1.docx]

**Supplementary Materials**

1. **Materials and Methods**

**1.1 HUVECs migration assay**

The migration ability of HUVECs under different conditions was analyzed by scratch test. In brief, 1 X 10^5^ cells per well were plated in 24-well plates and incubated at 37°C for 24 h until confluence. Next, a scratch was scraped with the tip of a sterile cell scraper (0.5mm, SPLScarTM). Cell debris was washed with PBS and then cultured in serum-free containing medium (SclenCell, USA) treated with PBS or sEVs (10^9^ particles/mL). Images of the wound were taken immediately, 3 h, and 7 h after scratching. We used Image J software to detect changes in the width of the scratch area.

**1.2 Tube formation assay of HUVECs**

HUVECs were seeded in 96-well plates at a density of 1.5 X10^4^ cells per well, and each seed well was already coated with 50 μL Matrigel (BD Biosciences, USA). The cells were treated with PBS or sEVs (10^9^ particles/mL) and then incubated at 37°C. After 4 h of culture, cells were imaged and analyzed using an inverted microscope (Olympus, Japan) and Image J software. Tube numbers, Nodes numbers, Master junction numbers and Total Length were measured.

**1.3 In vivo tracking of fluorescent sEVs**

Three groups of sEVs labeled with DID fluorescent dye (8µl per knee, 1x10^10^ particles/ml) were administered into the joint cavities of mice. Fluorescence tracing of the knee joints was conducted on days 1, 3, 5, and 7 post-injections using a small animal in vivo three-dimensional imaging system (IVIS SPECTRUM, PerkinElmer), and the max fluorescence intensity was quantified. On day 7, the mice were euthanized, and knee samples were harvested. Representative images of red fluorescence at the joints were captured using a stereomicroscope. Additionally, another set of joint samples underwent decalcification and made into frozen sections, and high-magnification fluorescence microscopy was used to capture representative images of red fluorescence from the cartilage layer and synovial tissue.

**1.4 Total Protein Extraction**

Primary cultured sEVs were lysed using lysis buffer (DB, 8M Urea, 100mM TEAB, pH 8.5). Cleavage products were performed by the following steps: centrifugation at 12,000g centrifugal force for 15 min at 4 °C. The supernatant was then treated with 1 mol/L DTT (Sigma, USA) for 1 h at 56 °C. Subsequently, it was alkylated with an appropriate amount of iodoacetamide for 1 h, followed by a brief ice bath for 2 min. The reaction was subsequently supplemented with an adequate quantity of IAM and incubated in a light-restricted environment at ambient temperature for 1 hour. The protein concentration was determined by measuring the absorbance of the samples at 595 nm using the Bradford Protein Quantification kit (Beyotime Biotechnology, China).

**1.5 Whole-proteome detection of sEVs**

UHPLC-MS/MS analyses were performed using a nanoElute UHPLC system (Bruker, Germany) coupled with a tims TOF pro2 mass spectrometer (Bruker, Germany) in Novogene Co., Ltd. (Beijing, China). Firstly, mobile phase A (100% water, 0.1% formic acid) and B solution (100% acetonitrile, 0.1% formic acid) were prepared. The lyophilized powder was dissolved in 10 μL of solution A, centrifuged at 14,000 g for 20 min at 4 °C, and 200 ng of the supernatant was injected into the Liquid chromatography-mass spectrometry system to detect. The model type of the UHPLC was nanoElute with nano-upgraded, and the analytical column was a home-made analytical column (15 cm×100 μm, 1.9 μm). The tims TOF pro2 mass spectrometry with Captive Spray ion source. spray voltage was set to 2.1 kV. The full scan range of the mass was from m/z 100 to 1700 and the Ramp time was 100 ms. The Lock Duty Cycle was set to 100%. The settings of PASEF were as following: 10 MS/MS scan (a total cycle time of 1.17 sec), ionic strength threshold of 2500, scheduling target intensity of 20,000. The raw data of MS detection was named as “.d”.

**1. 6 Proteomics Data Analysis**

All the resulting spectra were searched against homo_sapiens_uniprot_2023_3_13.fasta (207393 sequences) by the search engines: MaxQuant (Bruker, Tims). The protein quantitation results were statistically analyzed by a T-test. The proteins whose quantitation was significantly different among iChondrocyte-sEVs, iMSC-sEVs and iPSC-sEVs groups were defined as differentially expressed proteins based on P < 0.05 and fold-change (FC) >2 or FC < 0.5.

**2. Figures**


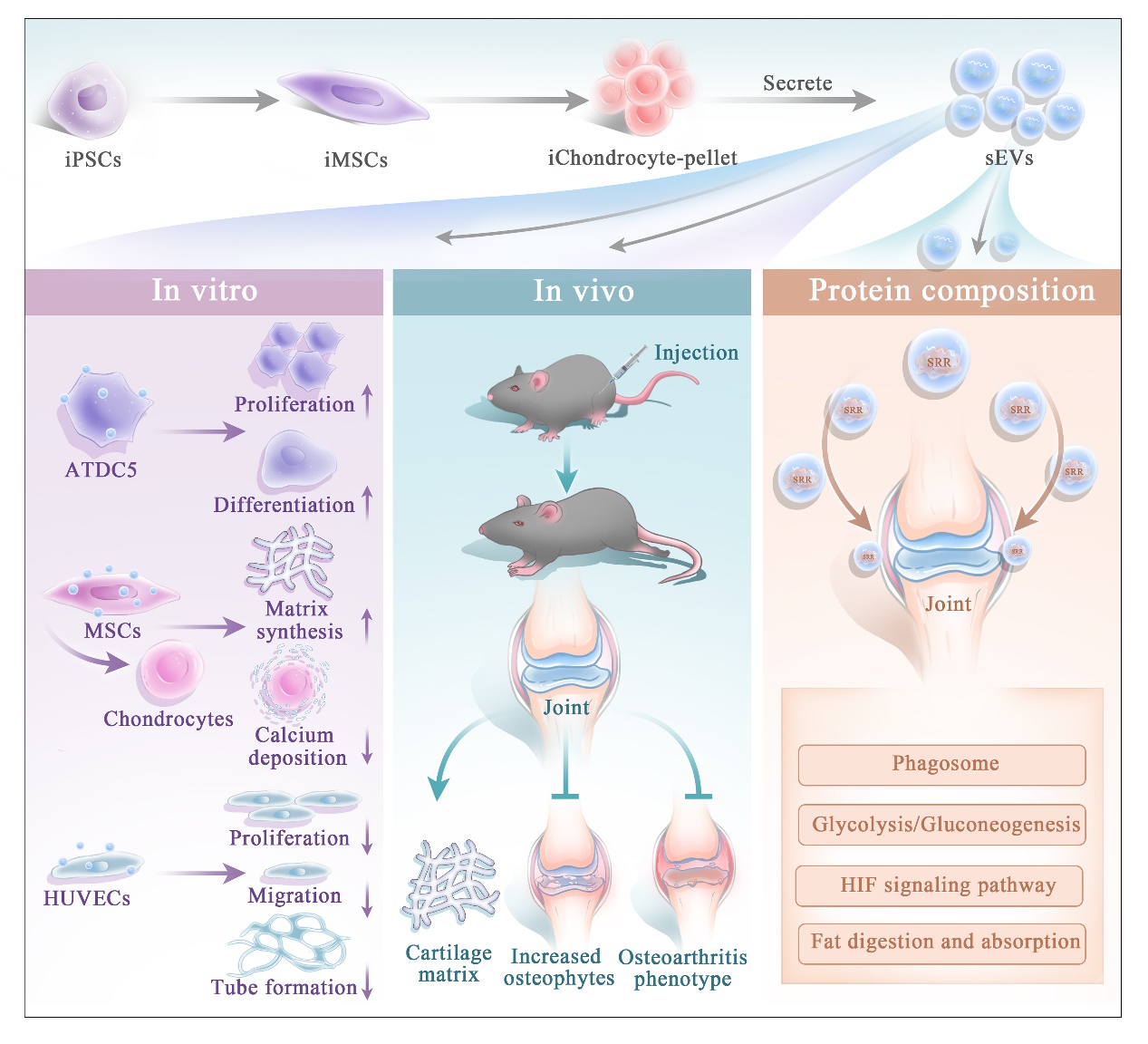


**Schematic diagram of the study.** The iChondrocyte-sEVs demonstrated significant enhancement of chondrogenic differentiation, promotion of extracellular matrix synthesis, and maintenance of chondrocyte homeostasis. In OA mice model, iChondrocyte-sEVs effectively mitigated multiple OA-related pathological features, restored cartilage microarchitecture and biomechanical properties, and markedly improved joint mobility. Comprehensive proteomic profiling of sEVs identified a distinct overexpression of SRR in iChondrocyte-sEVs. Furthermore, mechanistic investigations revealed the involvement of HIF-1, phagosome, glycolysis/gluconeogenesis, as well as fat digestion and absorption signaling pathways in the functional mechanisms of iChondrocyte-sEVs.


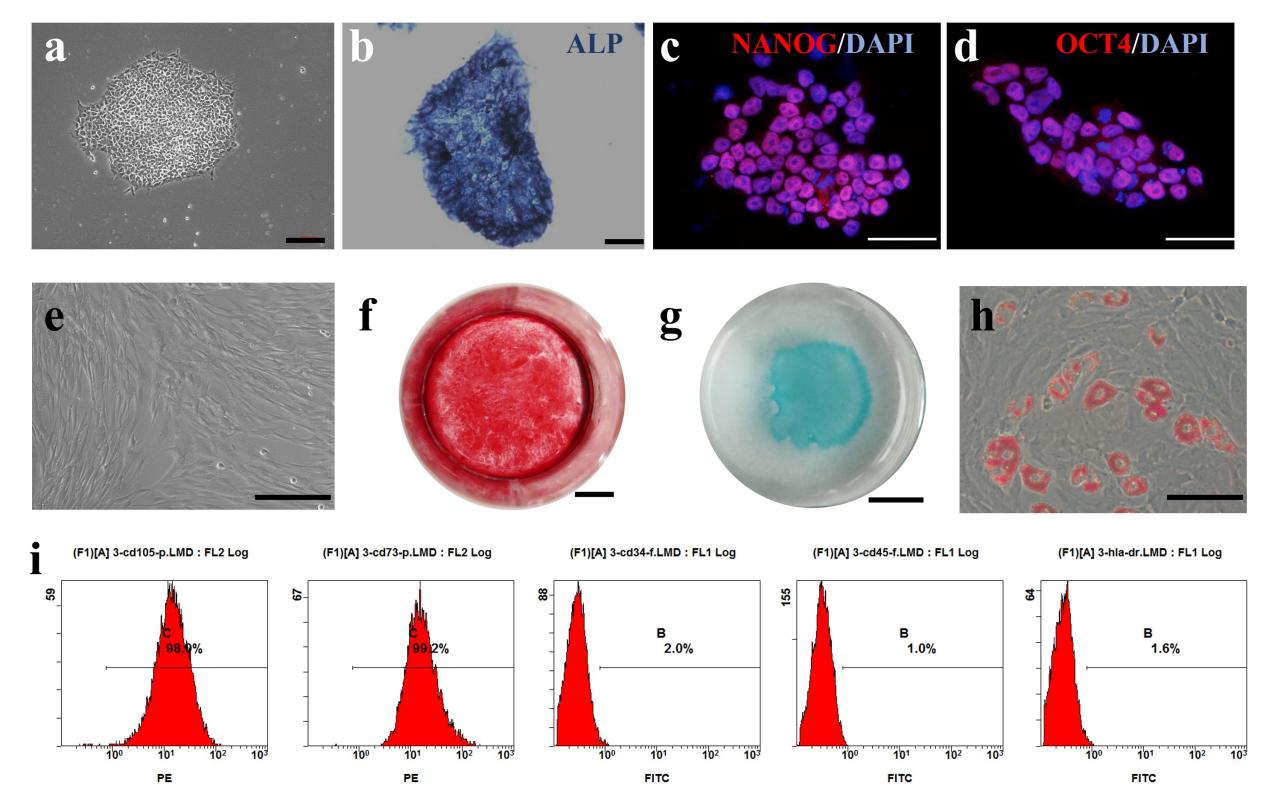


**Fig.S1 Characterization of iPSCs and iMSCs.** **a** Representative phase-contrast images demonstrating the typical embryonic stem cell-like morphology of iPSCs. **b** Alkaline phosphatase staining confirming the pluripotent status of iPSCs. **c-d** Immunofluorescence analysis of pluripotency markers in iPSCs, showing nuclear localization of NANOG (red) and OCT4 (red) with DAPI counterstaining (blue). **e** Phase-contrast micrographs illustrating the characteristic MSC-like morphology of iMSCs. **f-h** Histochemical staining demonstrating the trilineage differentiation potential of iMSCs: (**f)** Alizarin Red S staining for osteogenic differentiation, (**g)** Alcian Blue staining for chondrogenic differentiation, and (**h)** Oil Red O staining for adipogenic differentiation. **i** Flow cytometric analysis of iMSCs surface marker expression. The mesenchymal stem cell markers CD105 and CD73 were positively expressed, while hematopoietic markers CD34, CD45 and HLA-DR were negative. Data are presented as percentage of positive cells within the total cell population. Scale bars: a-e, h = 200 µm; f-g = 4 mm.


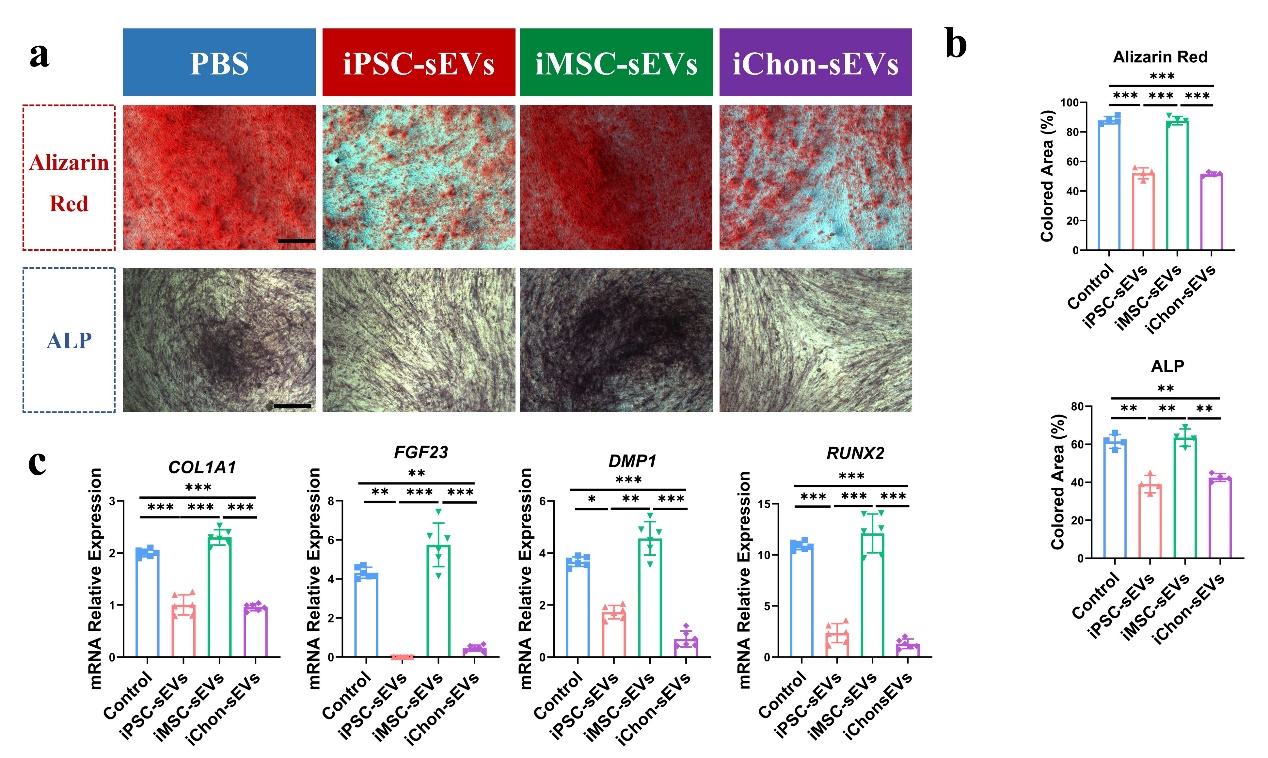


**Fig.S2 IChondrocyte-sEVs inhibited the differentiation of MSCs into osteoblasts.** **a** Representative images of Alizarin Red and ALP staining in MSCs undergoing osteogenic differentiation on day 21. Scale bar :200 um. **b** Quantitative analysis of the positively stained area for Alizarin Red and ALP in each experimental group (n=4). **c** Relative mRNA expression levels of osteogenic marker genes in MSCs during osteoblast differentiation (n=6). Data are presented as mean ± SD. **P* < 0.05, ***P* < 0.01, ****P* < 0.001.


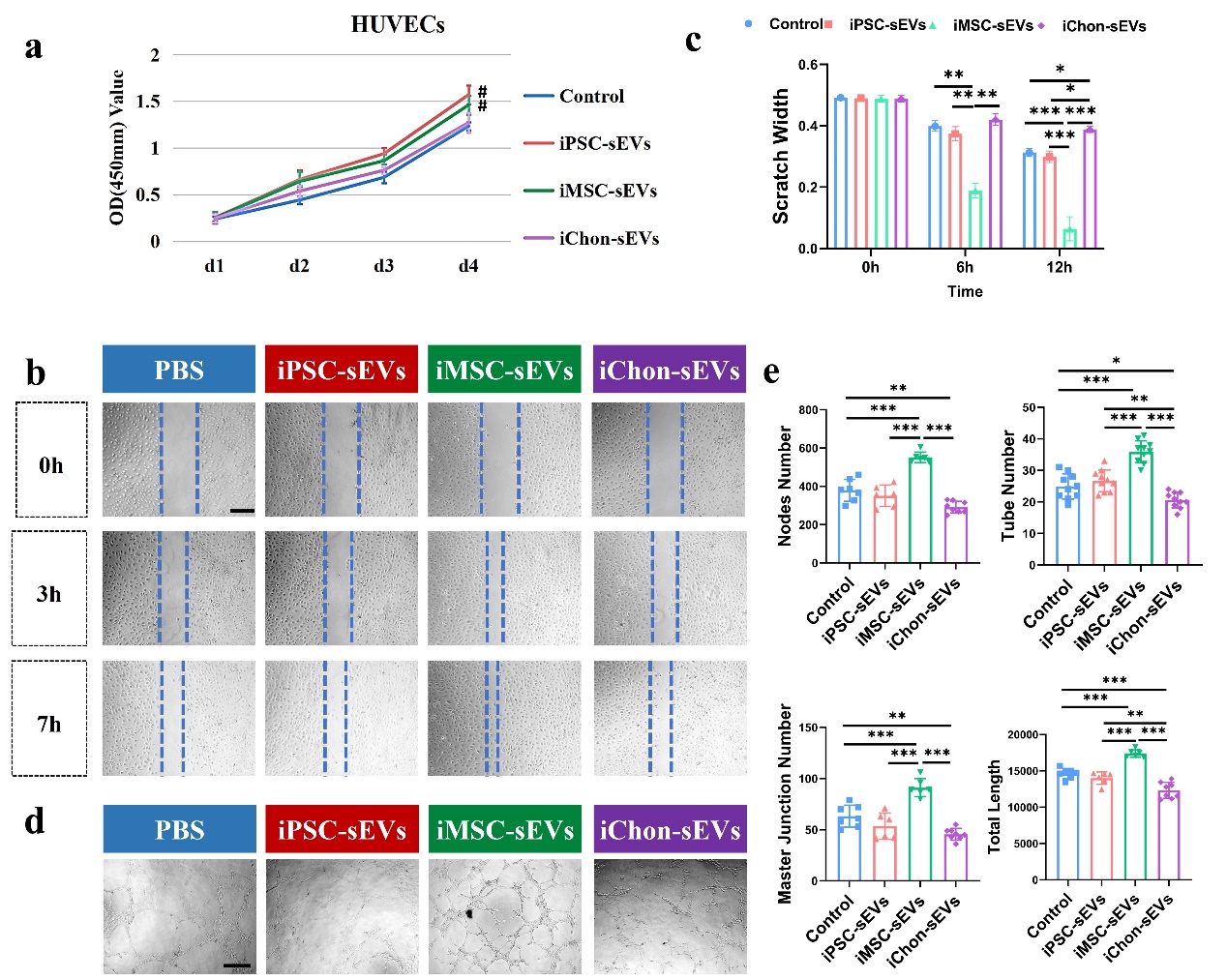


**Fig.S3 IChondrocyte-sEVs demonstrated significant inhibitory effects on proliferation, migration, and tube formation in HUVECs.** **a:** Proliferation kinetics of HUVECs monitored from day 1 to day 4 (n=6). **b** Time-course analysis of HUVECs migration using scratch wound assays at 0, 3, and 7-hour intervals (n=6). Scale bar: 200 µm. **c** Quantitative measurement of scratch wound closure (n=6). **d** Representative images from HUVECs tube formation assays. Scale bar: 200 µm. **e** Quantitative analysis of tube formation parameters, including tube number, nodes number, master junction number and total length (n=6). Data are presented as mean ± SD. **P* < 0.05, ***P* < 0.01, ****P* < 0.001; ^#^*P* < 0.05 vs. control group.


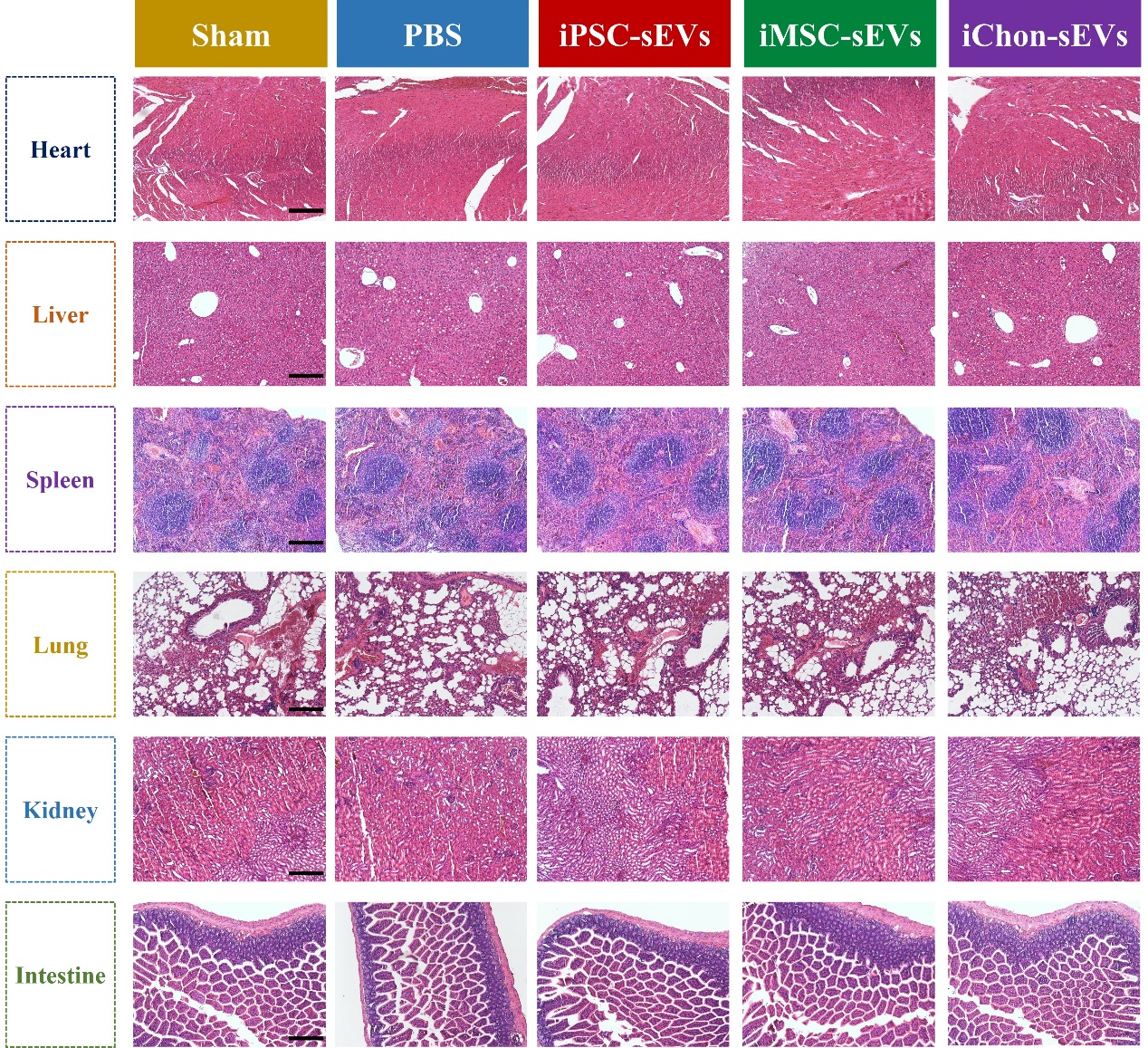


**Fig.S4 In vivo toxicity assays of sEVs.** Histopathological evaluation of mouse visceral organs was performed using H&E staining (n=6). Scale bar: 100 µm. All groups except the sham group were conducted under DMM conditions.


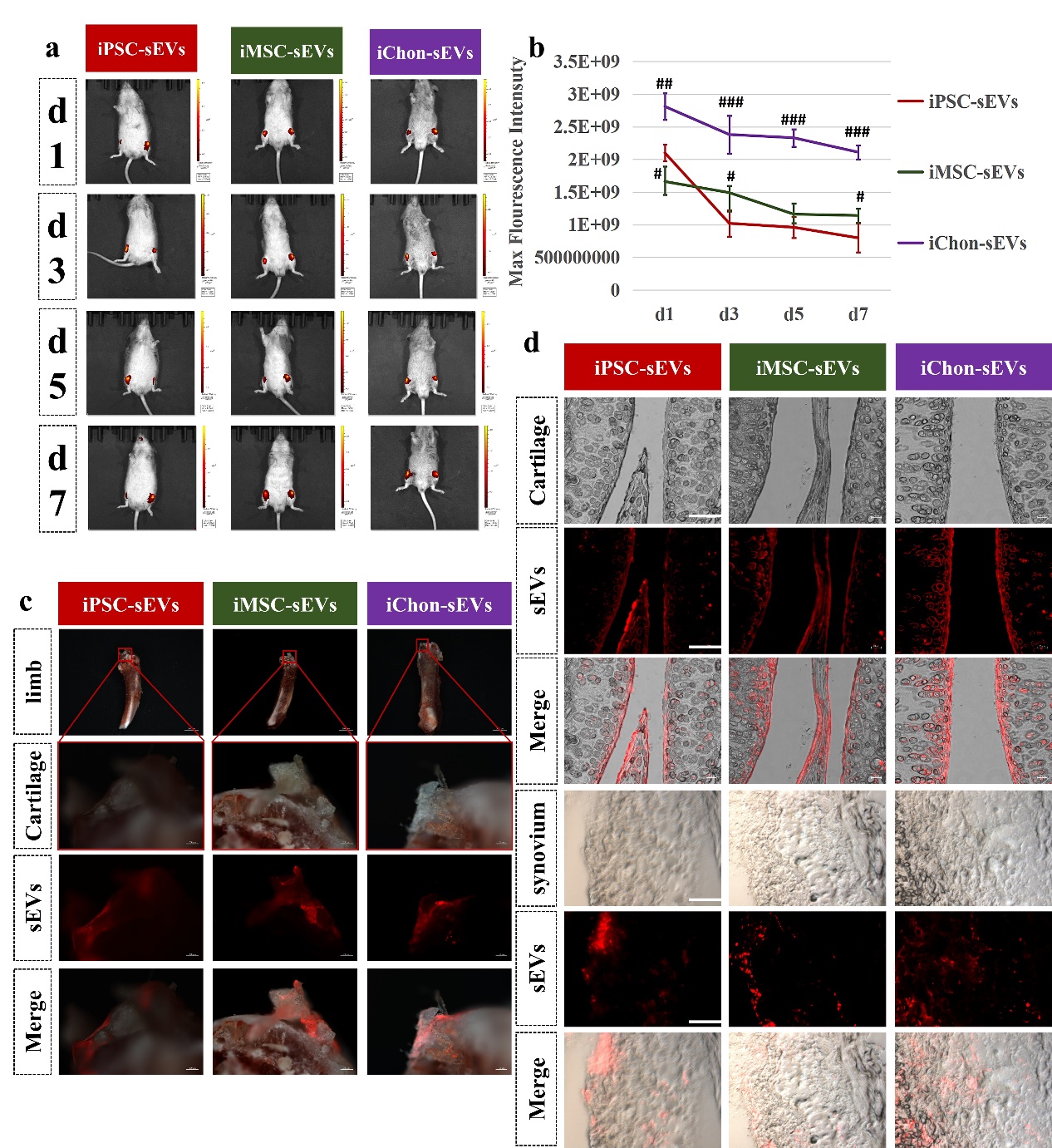


**Fig.S5 Penetration of sEVs into the articular cartilage layer.** **a:** Fluorescently labeled sEVs (8ul, 10^10^ particles/ml) were tracked within the joint cavity of mice from day 1 to day 7 (n=6). **b** Quantitative analysis of the maximum fluorescence intensity observed in the joint cavity of mice (n=6). **c** Fluorescent sEVs demonstrated colocalization with articular cartilage (n=6). Scale bars: 200 µm (top two rows), 100 µm (bottom two rows). **d** Fluorescent sEVs penetrated the cartilage layer and synovial tissue (n=6). Scale bar: 50 µm. Data are presented as mean ± SD. **^#^***P* < 0.05, **^##^***P* <0 .01 and **^###^***P* <0 .001 vs. iPSC-sEVs group.


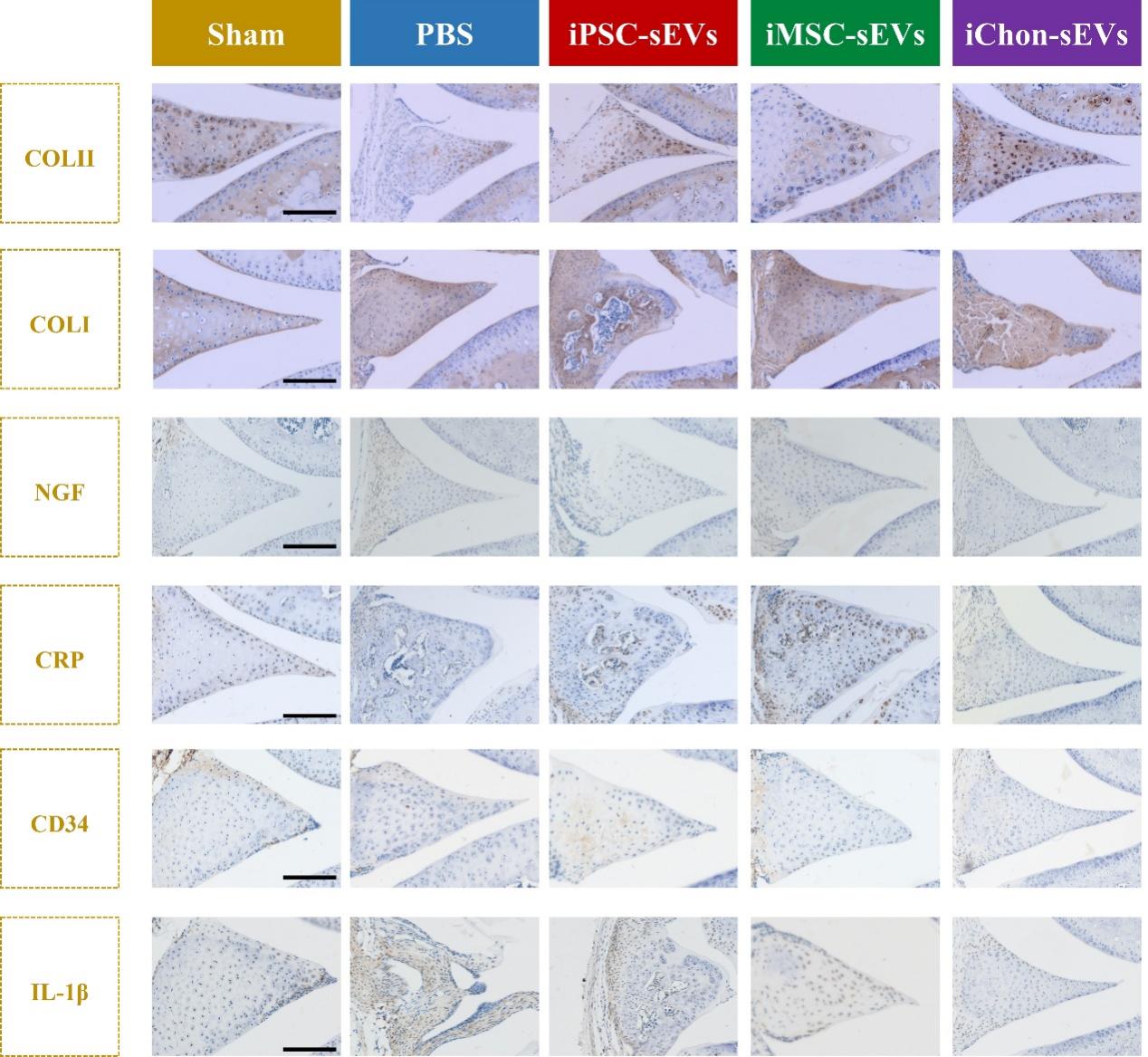


**Fig.S6 IChondrocyte-sEVs mitigated meniscal pathology in mice following DMM surgery.** Representative histopathological images illustrating key molecular markers associated with diverse OA phenotypes in the murine meniscus (n=6). Scale bar: 100 µm. All groups except the sham group were conducted under DMM conditions.
